# Functional framework of the kinetochore and spindle assembly checkpoint in Arabidopsis

**DOI:** 10.1093/plphys/kiaf461

**Published:** 2025-09-30

**Authors:** Aladár Pettkó-Szandtner, Zoltán Magyar, Shinichiro Komaki

**Affiliations:** Institute of Biochemistry, HUN-REN Biological Research Centre, Temesvári krt. 62, Szeged 6726, Hungary; Institute of Plant Biology, HUN-REN Biological Research Centre, Temesvári krt. 62, Szeged 6726, Hungary; Division of Biological Science, Nara Institute of Science and Technology, 8916-5 Takayama, Ikoma, Nara 630-0192, Japan

## Abstract

The kinetochore, critical for accurate chromosome segregation and genome stability in eukaryotes, comprises the Constitutive Centromere Associated Network (CCAN) and the KMN network. In animals, the CCAN associates with centromeric nucleosomes throughout the cell cycle, while the KMN network assembles at kinetochores during M phase, binding spindle microtubules and serving as a platform for the spindle assembly checkpoint (SAC) complex. Despite conserved functions, kinetochore components vary across organisms. In this study, we investigated the subcellular localization and interaction maps of core kinetochore components in Arabidopsis (*Arabidopsis thaliana*). Of the four conserved CCAN components, we found that only Centromere protein C (CENP-C) localizes to kinetochores, while all KMN components consistently localize to the kinetochore throughout the cell cycle. Immunoprecipitation assays revealed interactions between core kinetochore proteins and regulators involved in DNA replication, histone modification, and chromatin remodeling, suggesting that the kinetochore may also function outside of M phase. Examining interactions between kinetochore and SAC components allowed us to elucidate plant-specific SAC localization mechanisms, providing a functional framework for understanding plant kinetochores and offering insights into SAC regulation in plants.

## Teaser

In Arabidopsis, kinetochore analysis revealed plant-specific protein localizations and interactions, highlighting unique SAC regulation and suggesting kinetochore roles beyond chromosome segregation.

## Introduction

The kinetochore is a large protein complex essential for accurate chromosome segregation during cell division. The M phase checkpoint consisting of the Spindle Assembly Checkpoint (SAC) and the Chromosomal Passenger Complex (CPC; Lara-Gonzalez et al. 2012) monitors this process. The kinetochore, formed at the chromosome's centromere, comprises three layers: the inner centromere, the inner kinetochore, and the outer kinetochore.

The inner centromere is a specialized chromatin region between sister centromeres, where the CPC monitors interkinetochore tension and prevents chromosome missegregation (Carmena et al. 2012). The proteins that localize to the inner kinetochore throughout the cell cycle are known as the Constitutive Centromere-Associated Network (CCAN). In vertebrates, the CCAN consist of 16 proteins organized into five groups: CENP-C, CENP-H/I/K/M, CENP-L/N, CENP-O/P/Q/R/U, and CENP-S/T/W/X. CENP-C and CENP-N directly interact with CENP-A, a centromere-specific histone H3 variant, while CENP-T binds to centromere DNA. These interactions are essential for assembling the CCAN at the inner kinetochore (McAinsh and Meraldi 2011 ).

The outer kinetochore, extending from the inner kinetochore directly binds spindle microtubules. It consist of 10 core proteins forming three subcomplexes, collectively known as the KMN network: KNL1/ZWINT1 (KNL1 complex: KNL1C), MIS12/NNF1/NSL1/DSN1 (MIS12 complex: MIS12C), and NDC80/NUF2/SPC24/SPC25 (NDC80 complex: NDC80C; Varma and Salmon 2012). These subcomplexes facilitate spindle microtubule attachment and are crucial for force generation and signaling during chromosome movement.

The KMN network is recruited to the kinetochore via two independent pathways, mediated by CENP-C and CENP-T, in a sequential process throughout the cell cycle (Gascoigne et al. 2011). Initially, MIS12C and KNL1 are recruited to kinetochores during S phase (Gascoigne and Cheeseman 2013). NDC80 then localizes to the kinetochore following nuclear envelope breakdown (NEB) in the M phase. Mis12C directly binds to CENP-C (Screpanti et al. 2011), creating a binding site for NDC80 aiding in KMN network assembly. In parallel, CENP-T also directly binds to NDC80C, and facilitating the assembly of the KMN network (Nishino et al. 2013; Huisin’T Veld et al. 2016). These dual pathways have been proposed in several organisms, including Xenopus, chicken, and humans (Krizaic et al. 2015; Rago et al. 2015; Hara et al. 2018).

Although kinetochore functions are critical for chromosome segregation, a fundamental eukaryotic process, its composition varies significantly among organisms (Hooff et al. 2017). In *Caenorhabditis elegans* and *Drosophila melanogaster*, most CCAN components, including CENP-T, are absent. Thus, CENP-C alone mediates the CCAN and the KMN network interactions in these model organisms. Furthermore, many holocentric insects, early diverging fungi, and kinetoplastids lack CENP-A (Ishii and Akiyoshi 2022). Therefore, obtaining kinetochore information from more species is crucial to understand evolutionary changes in kinetochore factors and formation.

The SAC is a surveillance system that ensures equal chromosome segregation during cell division. Core SAC components, including BUBR1, BUB3, MAD1, MAD2, and the kinases BUB1 and MPS1, are conserved from yeast to animals (London and Biggins 2014a). Evolution has led to specialization and domain reshuffling among these core components such as BUB1-type proteins (Tromer et al. 2016). In a previous study, we found that Arabidopsis's BUB1-type paralogs, BMF1, BMF2, and BMF3, show domain reorganization and exhibit functional differences from animal BUB1 (Komaki and Schnittger 2017). Additionally, Arabidopsis BUB3.3 is more involved in chromosome congression than in the plant SAC (Lampou et al. 2023). Thus, despite SAC component conservation, SAC function and regulation likely vary among organisms.

The SAC complex is assembled on kinetochores unattached to microtubules. In yeast and humans, MPS1 kinase binds to NDC80, phosphorylates the MELT motifs in KNL1, and initiates SAC complex assembly at kinetochores (London et al. 2012; Shepperd et al. 2012; Yamagishi et al. 2012). The phosphorylated KNL1 facilitates the formation of the mitotic checkpoint complex, inhibiting the anaphase promoting complex/cyclosome (C) to prevent premature chromosome segregation.

Sequence analyses indicate that plant genomes have lost many CCAN components, but retain KMN components (Hooff et al. 2017; Yamada and Goshima 2017). In the moss *Physcomitrium patens*, only CENP-C (PpCENP-C) localizes to the kinetochore among conserved CCAN components, but it is absent post M phase, unlike in other organisms. Moss KMN components localize to kinetochores during M phase, similar to yeast and animals (Kozgunova et al. 2019). In flowering plants, the centromeric localization of CENP-C and KMN components (KNL1, MIS12, NDC80, NUF2, SPC24.1, and SPC25) through the cell cycle have been reported (Ogura et al. 2004; Sato et al. 2005; Shin et al. 2018; Li et al. 2021; Deng et al. 2024). In addition, several kinetochore components in flowering plants have been implicated in diverse cellular functions beyond chromosome segregation. For example, CENP-S and CENP-X are involved in limiting meiotic crossover, SPC24 promotes cell division, and NNF1 contributes to vegetative development (Girard et al. 2014; Shin et al. 2018; Allipra et al. 2022). However, comprehensive analyses are still lacking, leaving many aspects of plant kinetochore function and organization unclear. Previously, we showed that the SAC in Arabidopsis is rapidly deactivated during severe stress, impacting ploidy levels and plant evolution (Komaki and Schnittger 2017). However, the exact localization and regulation of the plant SAC at the kinetochore remained unknown.

This study provides a comprehensive localization and interaction map of core kinetochore components in Arabidopsis, detailing their assembly and functions beyond the M phase. We also identified unique plant-specific interactions between SAC and kinetochore factors, offering insight into the kinetochore structure and SAC regulation mechanism in plants.

## Results

### Spatiotemporal localization of core kinetochore components in Arabidopsis

**Figure 1.**
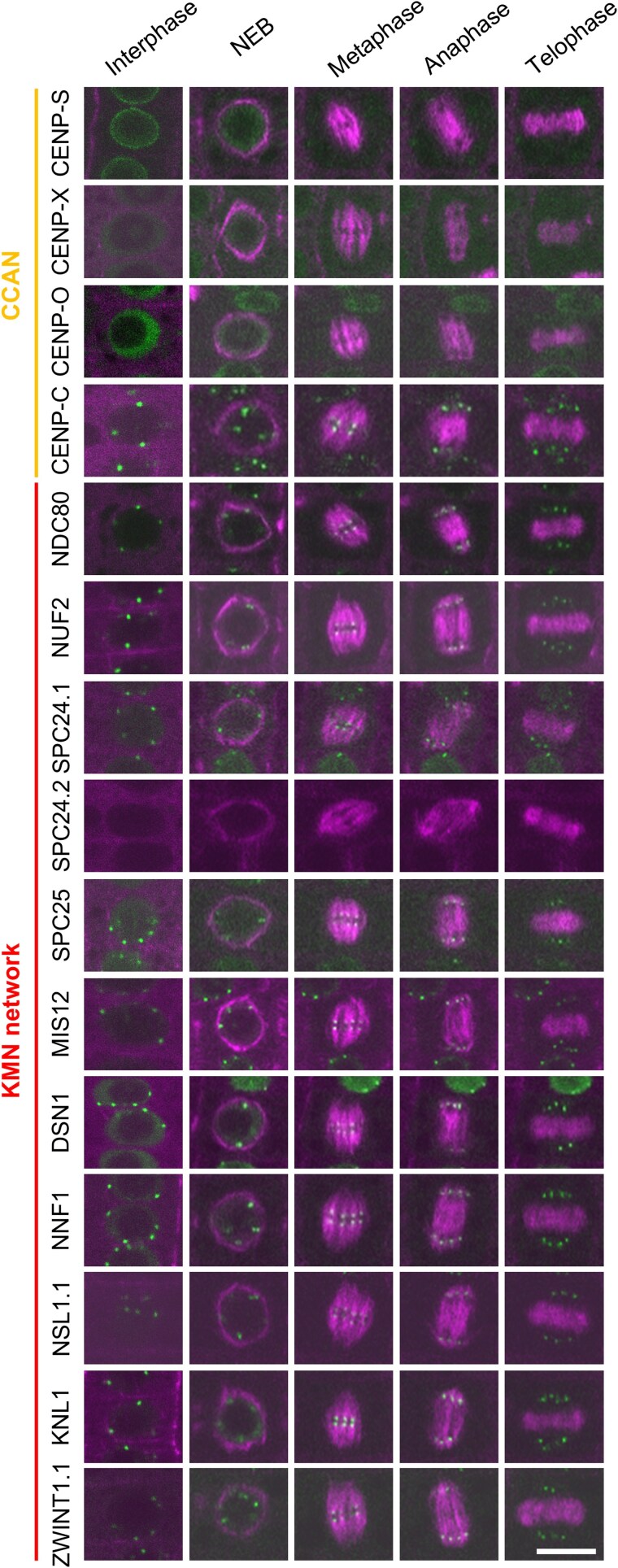
Subcellular localization of kinetochore component proteins during the cell cycle. Each kinetochore reporter line was crossed with TagRFP:TUA5 expressing plants to visualize microtubule structures. For live imaging, root tips of 5-day-old seedlings were used. NEB, nuclear envelope breakdown. Scale bar, 10 μm.

Genomic analysis identified four CCAN components and ten KMN network components in *Arabidopsis thaliana* (Table 1). Each component is conserved as a single gene, except for NSL1 (NSL1.1 and NSL1.2), SPC24 (SPC24.1 and SPC24.2) and ZWINT1 (ZWINT1.1 and ZWINT1.2). To investigate the spatiotemporal localization of these core kinetochore proteins, at least one gene family member was fused with the green fluorescent protein (GFP) coding sequence and transformed into Arabidopsis plants. The resulting lines revealed the localization patterns of these CCAN and KMN components in the root meristem (Fig. 1; Supplementary Videos S1 to S15).

**Table 1.** A comparative gene list of core kinetochore components in human and Arabidopsis

Human	Arabidopsis
CCAN	
CENP-C	AT1G15660
CENP-H	…
CENP-I	…
CENP-K	…
CENP-M	…
CENP-L	…
CENP-N	…
CENP-O	AT5G10710
CENP-P	…
CENP-Q	…
CENP-R	…
CENP-U	…
CENP-S	AT5G50930
CENP-X	AT1G78790
CENP-T	…
CENP-W	…
KMN network	
KNL1	AT2G04235
ZWINT1	ZWINT1.1: AT3G23910 ZWINT1.2: AT3G24255
MIS12	AT5G35520
DSN1	AT3G27520
NSL1	NSL1.1: AT4G00525 NSL1.2: AT1G01715
NNF1	AT4G19350
NDC80	AT3G54630
NUF2	AT1G61000
SPC24	SPC24.1: AT3G08880 SPC24.2: AT5G01570
SPC25	AT3G48210

In animals, the CCAN components localize to kinetochores throughout the cell cycle. However, among the conserved CCAN components in Arabidopsis, only CENP-C localized to kinetochores during both interphase and mitosis, consistent with previous findings in the moss, *P. patens* (Kozgunova et al. 2019; Fig. 1; Supplementary Videos S1 to S4). In contrast, CENP-S, CENP-X, and CENP-O did not localize to kinetochores at any stage of the cell cycle. CENP-S was observed at the nuclear envelope during interphase and relocated to the cytoplasm during mitosis (Fig. 1; Supplementary Video S1). CENP-X and CENP-O remained cytoplasm throughout the cell cycle (Fig. 1; Supplementary Videos S2 to S3), suggesting that these proteins may not function as canonical kinetochore components in Arabidopsis. Supporting this, it has been reported that mutants of core kinetochore components show lethal or severe growth defects (Sato et al. 2005; Girard et al. 2014; Shin et al. 2018; Allipra et al. 2022; Deng et al. 2024), whereas *cenp-s* and *cenp-x* mutants display no apparent phenotype during the vegetative stage. As the T-DNA line of *cenp-o-1* (SALK_149069) was able to express the full-length *CENP-O* transcript, we generated a CRISPR/Cas9-induced mutant to investigate its function (Supplementary Fig. S1). The resulting *cenp-o-2* allele carried a 7-bp deletion that introduced a premature stop codon; nevertheless, the mutant also exhibited no obvious phenotypes, reinforcing the idea that these three CCAN components may not play essential roles in kinetochore function in Arabidopsis.

The KMN network, composed of KNL1C, MIS12C, and NDC80C, is localized to the outer kinetochore. In animal cells, the KMN network is sequentially recruited to the kinetochore throughout the cell cycle, with all components assembling only during M phase. Although SPC24.2 was not detected in root cells, other KMN components were localized to kinetochores during both interphase and mitosis (Fig. 1; Supplementary Videos S5 to S15).

In summary, only CENP-C exhibited the typical localization pattern of CCAN components in Arabidopsis. Furthermore, all KMN components in Arabidopsis are consistently localized to the kinetochore, unlike in animals.

### Building the Arabidopsis core kinetochore interactome

**Figure 2.**
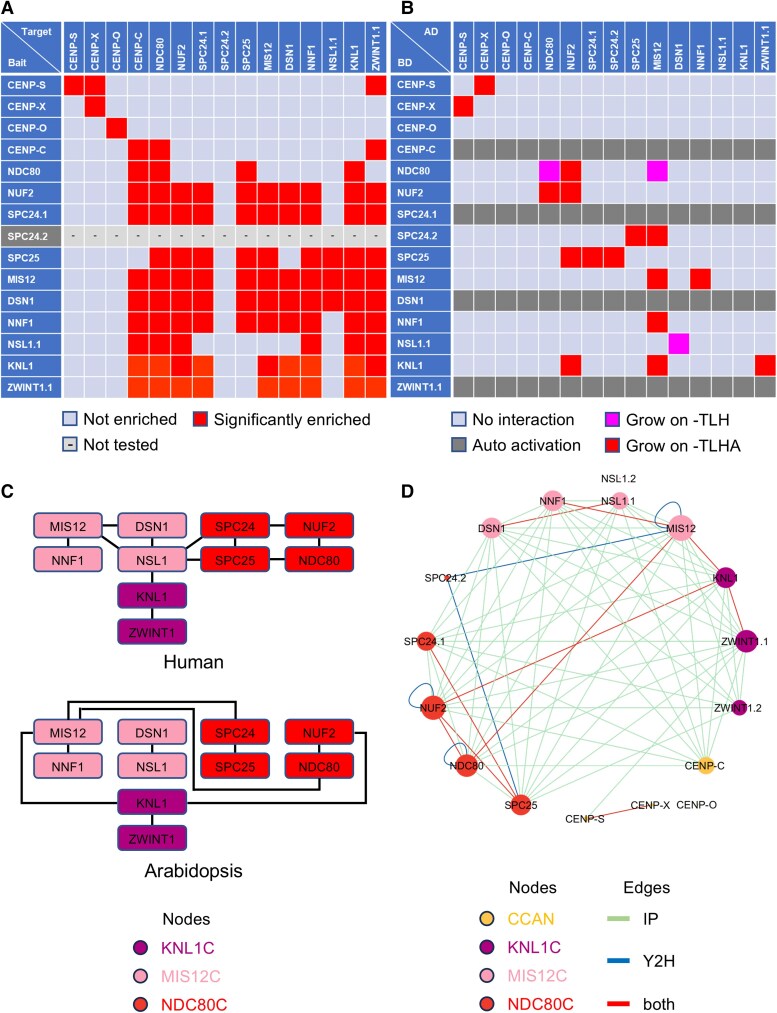
Interaction map of Arabidopsis core kinetochore components. **A)** Interaction among the core kinetochore components as revealed by IP. SPC24.2:GFP was not utilized in IP experiments, as no expression was observed in seedings. The corresponding IP data is available as Supplementary Table S1. **B)** Results of Y2H assays among core kinetochore components. Each yeast strain was spotted on SD plates without Trp and Leu (−TL; control media), without Trp, Leu, and His (−TLH; medium selection media), or without Trp, Leu, His, and Ade (−TLHA; severe selection media), and photographed after incubation at 30 °C for 3 days. The interactions were classified according to yeast growth on different selection media in three categories. AD, GAL4-activation domain; BD, GAL4-DNA binding domain. The corresponding Y2H data is available as Supplementary Fig. S2. **C)** Comparison of binding patterns between KMN network factors in human and Arabidopsis. The binding model for Arabidopsis is based on Y2H results but it does not account for homodimerization. **D)** Cytoscape representation of the observed interaction network. The color and size of a node represent the subcomplex to which it belongs, and the rate of occurrence of each protein, respectively. The edge colors indicate the type of assay that detected the interaction: IP (green), Y2H (blue), and both methods (red).

To elucidate the interaction network of core kinetochore components in Arabidopsis, we conducted immunoprecipitation (IP) experiments using an antibody against GFP on all kinetochore-GFP lines, except SPC24.2:GFP, followed by mass spectrometry. Each bait was purified at least twice from extracts of 1-week-old seedlings. We identified bait-specific interactions by selecting proteins with at least two unique peptides detected in at least two independent experiments (see Materials and Methods for details). Potential background proteins were excluded based on their occurrence in the 10 negative controls, resulting in 863 unique proteins as potential interactors of the 14 Arabidopsis core kinetochore components (Fig. 2A and C; Supplementary Table S1). GO enrichment analysis revealed that the IP lists were over 10-fold enriched in proteins linked to “kinetochore organization” (31.98-fold; *P* = 2.57E−03) and “centromere complex assembly” (15.99-fold; *P* = 1.77E−02; Supplementary Table S2).

To identify direct interactions among the IP results, we conducted a Y2H assay, testing 256 binary interactions among core kinetochore components in Arabidopsis. The cDNA of all kinetochore components and GFP, as a negative control, were cloned in pGBT9 (bait) and pGAD424 (prey) vectors and cotransformed in yeast cells with all 256 combinations (Fig. 2B and C; Supplementary Fig. S2). Since SPC24.2 cDNA was successfully amplified from flowering tissue, it was included in the Y2H assay. Interactions were assessed by measuring growth on control (−TL), moderately selective (−TLH), and severely selective (−TLHA) media. However, CENP-C, SPC24.1, DSN1, and ZWINT1.1 with DB domain-containing constructs exhibited self-activation complicating the evaluation of their interactions.

### A comprehensive interaction map of the core kinetochore

We first examined the core kinetochore proteins. In yeast and human cells, the CCAN is constantly localized to kinetochores and is essential for the formation of the KMN network (Hara and Fukagawa 2018). In Arabidopsis, only CENP-C among the CCAN components localized to kinetochores (Fig. 1), copurifying with many KMN components. However, other CCAN components like CENP-S, CENP-X, and CENP-O, were rarely detected in our IP samples (Fig. 2A and D). Only the CENP-S and CENP-X interaction was observed by both IP and Y2H (Fig. 2A, B and D).

Conversely, KMN network components were frequently detected in each other's IP samples indicating a robust complex (Fig. 2A and D). Y2H results showed that the KNL1C, MIS12C, and NDC80C subcomplexes directly bound to each other through at least one component (Fig. 2B and C). However, the bridging components differed from animals, where NDC80C interacts with NSL1 of the MIS12C via SPC24-SPC25 (Petrovic et al. 2010; Fig. 2B and C). In Arabidopsis, NDC80C interacts with MIS12 via NDC80 and SPC24 (Fig. 2B and C). In animals, the KNL1C localizes to the kinetochore through KNL1 and MIS12C's NSL1 (Petrovic et al. 2010). In Arabidopsis, KNL1 binds to ZWINT1.1, MIS12 and NUF2 indicating localization via both MIS12C and NDC80C subcomplexes (Fig. 2B and C). Similar to animals, Arabidopsis MIS12C components formed MIS12-NNF1 and NSL1.1-DSN1 heterodimers (Fig. 2B and C). Additionally, Y2H results showed homodimerization of NDC80, NUF2, and MIS12 (Fig. 2B). Collectively, subcellular localization results indicate that the Arabidopsis KMN network forms a complex at the kinetochore throughout the cell cycle, exhibiting distinct binding modes compared to animals.

**Figure 3.**
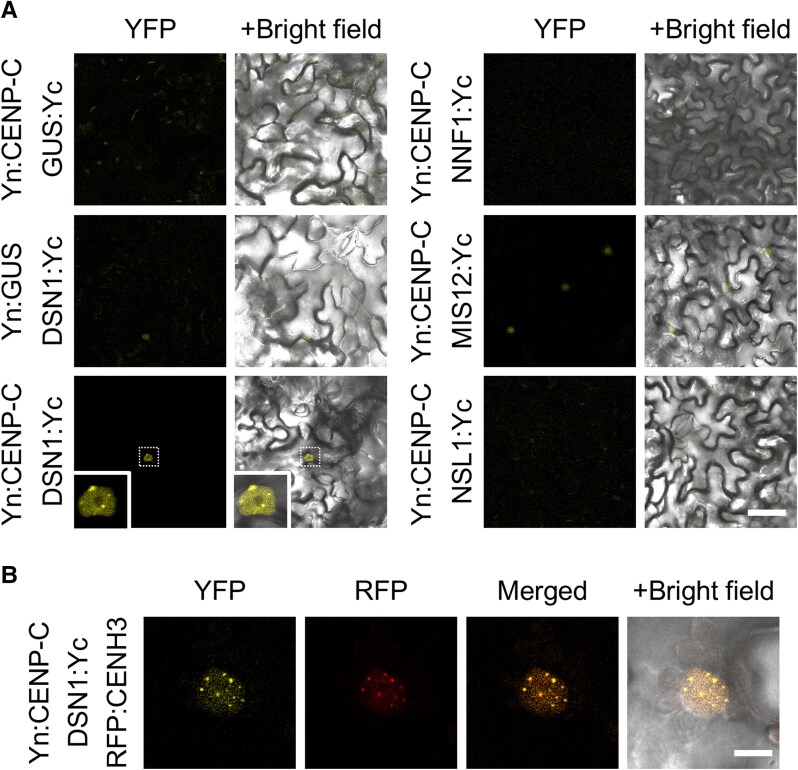
Interaction between CENP-C and MIS12 complex. Analysis of the interaction between CENP-C and MIS12C proteins in *N. benthamiana* by BiFC assay. **A)** CENP-C was fused to the N-terminal fragment of YFP (Yn), while each of the four MIS12C proteins was fused to the C-terminal fragment of YFP (Yc). The GUS protein served as a negative control. Boxed regions are shown at higher magnification (4×) to highlight YFP fluorescence signals. Scale bar, 50 μm. **B)** Colocalization assay of reconstituted YFP signals (Yn:CENP-C and DSN1:Yc) with the centromere marker RFP:CENH3. Scale bar, 10 μm.

In both yeast and human cells, CENP-C plays a central role in organizing the KMN network at the kinetochore through its direct interaction with MIS12C. Our IP results suggest that Arabidopsis CENP-C similarly contributes to KMN network recruitment via its association with MIS12C. However, because Arabidopsis CENP-C exhibited self-activation in our Y2H assay, we turned to bimolecular fluorescence complementation (BiFC) assays to detect direct interactions between CENP-C and MIS12 complex (Fig. 3A). For this purpose, the candidate proteins were tagged with the N-terminal and C-terminal fragments of Yellow Fluorescent Protein (YFP) and coinfiltrated into *Nicotiana benthamiana* leaves. As shown in Fig. 3A, a YFP signal was detected exclusively when CENP-C and DSN1 were combined. The reconstituted YFP formed distinct dot-like signals within the nucleus, suggesting that the interaction occurs at the kinetochores (Fig. 3A). This interpretation is supported by the colocalization of the YFP signals with the centromere marker RFP:CENH3 (Fig. 3B). These findings indicate that the CENP-C–DSN1 interaction serves as a critical bridge between CCAN and KMN in Arabidopsis.

### Identification of interactors with core kinetochore components in Arabidopsis

Our IP data set revealed numerous proteins previously unidentified as kinetochore interactors in plants. Focusing on cell cycle and/or cytoskeleton-related proteins under specific GO IDs: cell cycle (GO:0007049), cell division (GO:0051301), cell population proliferation (GO:0008283), chromosome segregation (GO:0007059), DNA replication (GO:0006260), chromosome organization (GO:0051276), histone binding (GO:0042393), chromatin organization (GO:0006325), cytoskeleton organization (GO:0007010), and cytoskeletal protein binding (GO:0008092). Among 1,712 Arabidopsis proteins 98 related to the cell cycle and cytoskeleton were identified in the IP samples (Supplementary Table S3).

CDKA;1, a key cyclin-dependent kinase (CDK) in Arabidopsis, copurified with core kinetochore proteins. KNL1, NUF2, and DSN1 were phosphorylated at [S/T]P CDK consensus sites (Supplementary Table S4), suggesting that CDKA;1 may be responsible for these modifications. The IP lists also include DNA replication factors (PCNA1, PCNA2, RFC3, RPA2, FEN1, and MCM3) and histone modification proteins (HDA6, HDA19, HOS15, and WDR5A), indicating a linkage between kinetochores and DNA replication. NASP, recently identified as the CENH3 chaperone in plants (Le Goff et al. 2020; Takeuchi et al. 2024), is also present in the IP lists, implying a role for kinetochore in CENH3 deposition. Additionally, subunits of the SWI/SNF complex (SWI3A, SWI3B, SWI3C, SWI3D, and BSH) were frequently observed. The SWI/SNF complexes, known for chromatin remodeling and transcription regulation, have also been localized to yeast kinetochores (Xue et al. 2000), suggesting a similar role in plants. Moreover, several kinesins (KINUB, KIN4A, KIN13A, KIN13B, and KIN14E) and microtubule-binding proteins (MBP2C, SPR2, WDL2, and MAP65-2) copurified with core kinetochore components, interacting with cortical microtubules and regulating cell morphology, thus emphasizing kinetochores' broader functional significance beyond the M phase.

### SAC assembly at kinetochore

**Figure 4.**
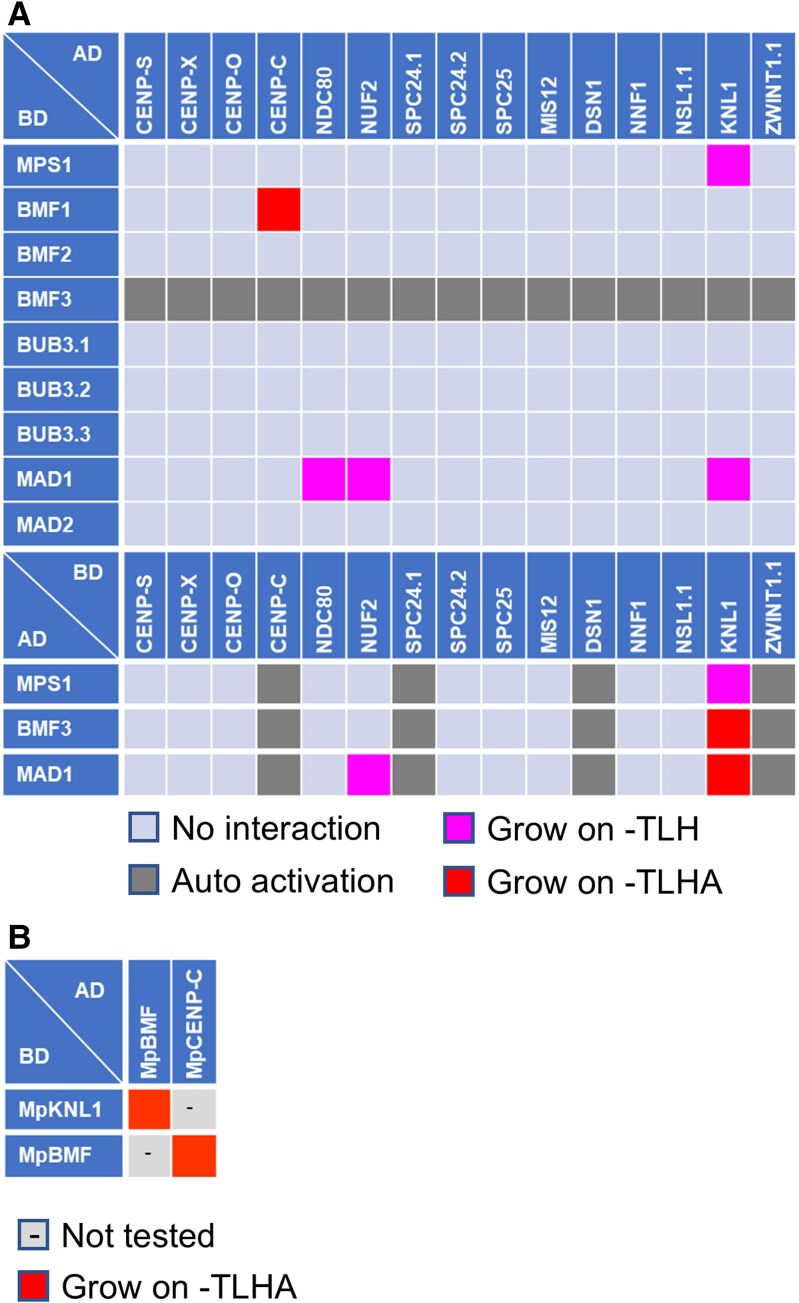
Interaction between core kinetochore and SAC components. **A)** Results of Y2H assays between Arabidopsis core kinetochore components and SAC components. **B)** Results of Y2H assays between *M. polymorpha* core kinetochore components (MpCENP-C and MpKNL1) and SAC component (MpBMF). Each yeast strain was spotted on SD plates without Trp and Leu (–TL; control media), without Trp, Leu, and His (–TLH; medium selection media), or without Trp, Leu, His, and Ade (-TLHA; severe selection media), and photographed after incubation at 30 °C for 3 days. The interactions were classified according to yeast growth on different selection media in three categories. AD, GAL4-activation domain; BD, GAL4-DNA binding domain. The corresponding Y2H data are available as Supplementary Fig. S3.

The SAC complex localizes to kinetochores, ensuring genome stability by facilitating proper chromosome segregation during mitosis and meiosis. In fission yeast and human cells, kinase MPS1, a SAC component, localizes to kinetochores via NDC80, phosphorylates KNL1 and activates SAC signaling (Yamagishi et al. 2012; London and Biggins 2014a). SAC silencing involves the competition for NDC80 binding between spindle microtubules and MPS1 (Hiruma et al. 2015; Ji et al. 2015). Therefore, the MPS1-NDC80 interaction is vital for both SAC activation and silencing. Our previous work showed that the Arabidopsis SAC components MPS1, BMF1, BMF3, MAD1, and MAD2 are present at kinetochores (Komaki and Schnittger 2017). However, the specific kinetochore factors responsible for recruiting these SAC components remained unclear. We explored this by analyzing SAC and conserved kinetochore component interactions using Y2H assay (Fig. 4A; Supplementary Fig. S3). Surprisingly, Arabidopsis MPS1 binds directly to KNL1 but not to NDC80 (Fig. 4A), suggesting that, unlike in some other systems, MPS1 does not compete with microtubules for NDC80 binding in Arabidopsis cells. However, we were unable to detect MPS1 binding to either KNL1 or NDC80 in BiFC assays, indicating that further studies are needed to validate and clarify the nature of these interactions (Supplementary Fig. S4).

In human cells, MPS1-phosphorylated KNL1 scaffolds BUB3-BUB1 and BUB3-BUBR1 complexes recruiting the MAD1-MAD2 complex to kinetochores through BUB1 kinase and MAD1 interaction mediated by a conserved domain 1 (CD1) in BUB1 (London and Biggins 2014b). In Arabidopsis BUB1 and BUBR1 functions are divided among three proteins: BMF1 (BUB1 kinase domain), BMF2 (BUBR1-like), and BMF3 (BUB1 CD1-like domain; Komaki and Schnittger 2017). We demonstrated that BMF3 interacts with MAD1 via its CD1-like domain, and Arabidopsis MAD1 also binds core kinetochore components, including NDC80, NUF2, and KNL1 (Fig. 4A). Thus, the MAD1-MAD2 complex may localize to the kinetochore via BMF3-dependent and independent pathways.

**Figure 5.**
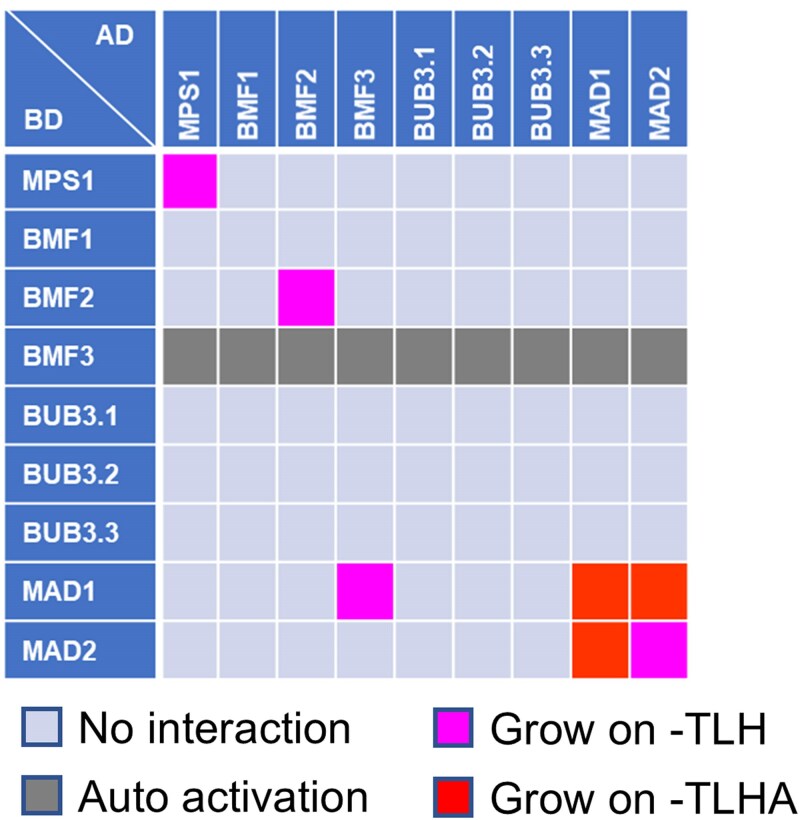
Interaction among Arabidopsis SAC components. Results of Y2H assays among Arabidopsis SAC components. Each yeast strain was spotted on SD plates without Trp and Leu (–TL; control media), without Trp, Leu, and His (–TLH; medium selection media), or without Trp, Leu, His, and Ade (–TLHA; severe selection media), and photographed after incubation at 30 °C for 3 days. The interactions were classified according to yeast growth on different selection media in three categories. AD, GAL4-activation domain; BD, GAL4-DNA binding domain. The corresponding Y2H data are available as Supplementary Fig. S5.

To map SAC complex interactions, we investigated SAC component binding. Besides previously identified interactions (MAD1-BMF3, MAD1-MAD1, and MAD1-MAD2; Komaki and Schnittger 2017), we found that MPS1 and BMF2 form homodimers (Fig. 5; Supplementary Fig. S5). These findings provide a more detailed understanding of SAC assembly and kinetochore regulation.

### KNL1 functions as an interaction hub between the kinetochore and the SAC

**Figure 6.**
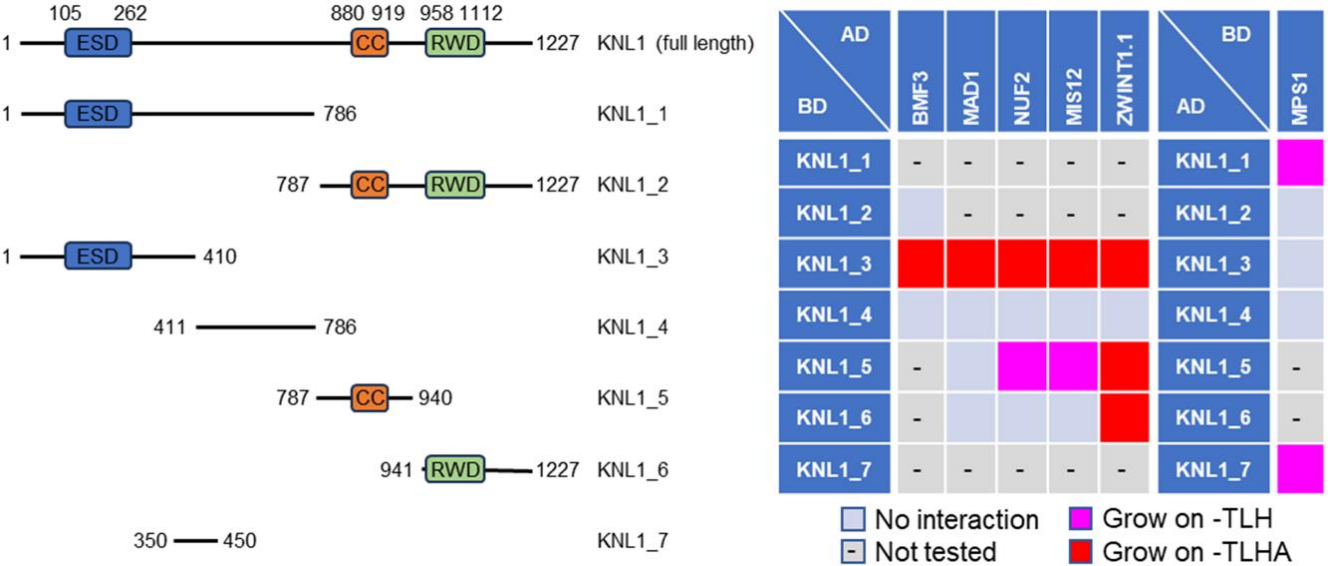
Identification of the interaction domain between KNL1 and its interactors. Y2H assays with the KNL1 fragments and its interactors. Each yeast strain was spotted on SD plates without Trp and Leu (–TL; control media), without Trp, Leu, and His (–TLH; medium selection media), or without Trp, Leu, His, and Ade (–TLHA; severe selection media), and photographed after incubation at 30 °C for 3 days. The corresponding Y2H data are available as Supplementary Fig. S6.

Our data showed that three kinetochore components (MIS12, NUF2, and ZWINT1.1) and three SAC components (BMF3, MAD1, and MPS1) bind to KNL1 in plants (Figs. 2B and 4A). To identify KNL1's interaction domain, we examined truncated versions of the KNL1 construct using Y2H assay. Arabidopsis KNL1 has a eudicot-specific-domain (ESD), coiled-coil (CC), and RING-WD40-DEAD (RWD) domains (Deng et al. 2024). BMF3 binds to the ESD containing region, as previously demonstrated (Fig. 6; Supplementary Fig. S6; Deng et al. 2024), while MAD1 and MPS1 bind to the ESD-containing region and the region spanning amino acids 350 to 450, respectively (Fig. 6; Supplementary Fig. S6). Although this MPS1-binding region in KNL1 is not well conserved at the amino acid sequence level across plant species, structural prediction using AlphaFold 2 and 3 suggest that these regions form helical conformations (Supplementary Fig. S7A and B; Varadi et al. 2024). In animals, kinetochore components, ZWINT1 and NSL1, bind to the CC and the RWD domains, respectively (Kiyomitsu et al. 2011; Petrovic et al. 2014). Our results demonstrated that Arabidopsis NUF2, MIS12, and ZWINT1.1 bind to the ESD containing region and the CC domain with ZWINT1.1 also binding to the RWD domain (Fig. 6; Supplementary Fig. S6).

These results indicate that KNL1 acts as a hub connecting kinetochores and the SAC through various pathways.

### BMFs loading system at the kinetochores

Su et al. (2021) have shown that maize KNL1 (ZmKNL1) binds to ZmBMF1 and ZmBMF2. In contrast, Arabidopsis KNL1 binds to BMF3, suggesting distinct BMFs loading system at the kinetochores in monocots and eudicots (Deng et al. 2024). Our Y2H results supports that Arabidopsis BMF3 binds to KNL1. Additionally, Arabidopsis BMF1, which has a BUB1 kinase domain, binds to CENP-C (Fig. 4A). Chlorophyta, such as *Micromonas pusilla* and *Volvox carteri*, possess a single BMF gene (Vleugel et al. 2012). To explore the evolutionary aspect of BMFs loading systems, we studied the moss *Marchantia polymorpha*, an early diverging land plant model identifying one *BMF* family gene (*MpBMF*) that binds both MpCENP-C and MpKNL (Fig. 4B; Supplementary Fig. S3). This suggests the original plant BMF could bind to both proteins with functions splitting into BMF1 and BMF3 in angiosperms, each developing distinct binding partners.

## Discussion

**Figure 7.**
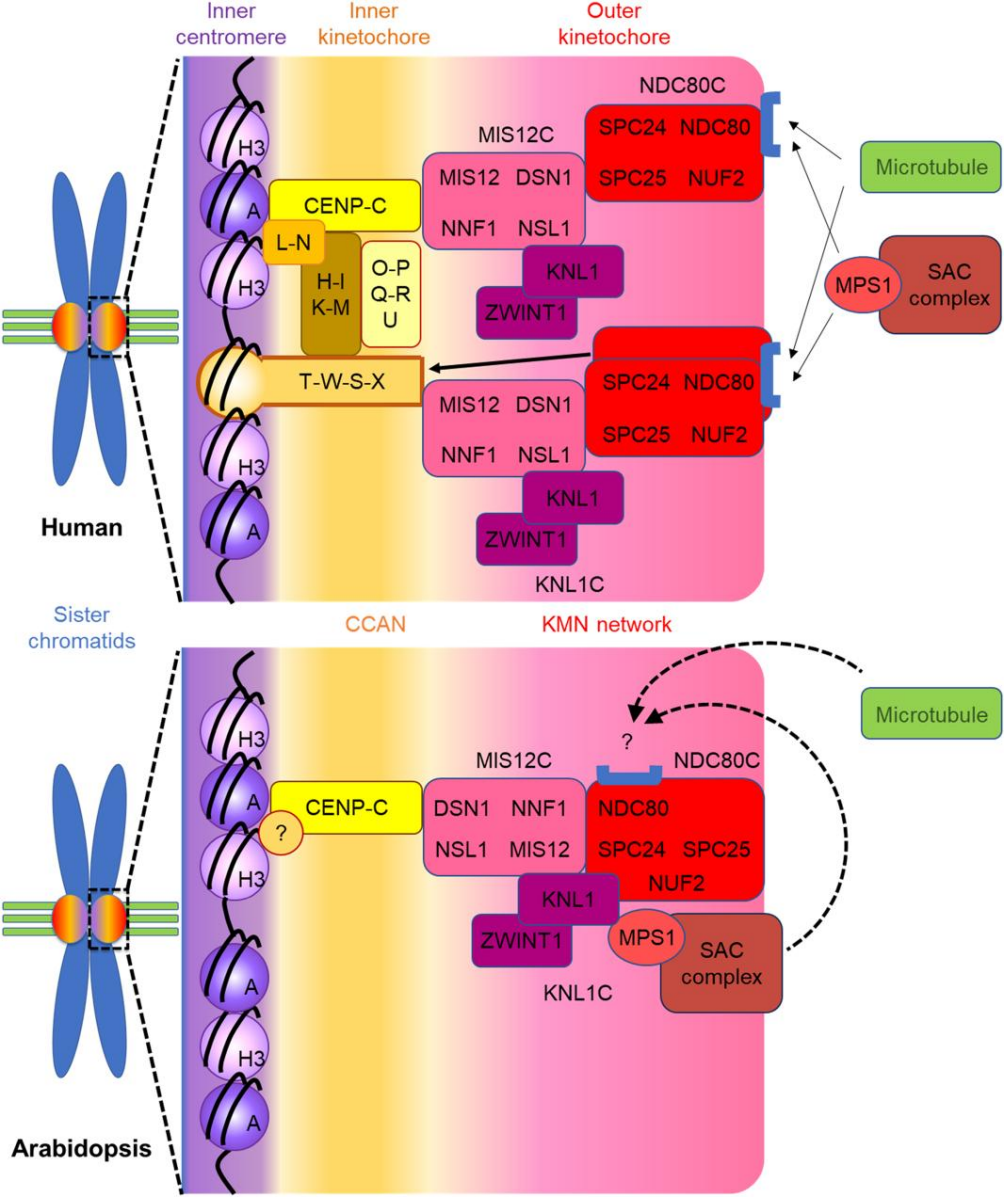
A schematic comparison of the interactions between core kinetochore and SAC components in human and Arabidopsis. In humans, the CCAN at the inner kinetochore recruits the KMN network through direct interactions with CENP-T and CENP-C. In Arabidopsis, the interaction between CENP-C and DSN1, a component of the MIS12 complex, likely plays a central role in connecting the CCAN and KMN networks. However, it remains unclear whether Arabidopsis CENP-C can directly interact with CENH3. In human cells, MPS1 and spindle microtubules compete for the same binding region on NDC80, a mechanism that contributes to spindle assembly checkpoint (SAC) silencing. In contrast, Arabidopsis MPS1 appears to bind KNL1 rather than NDC80, suggesting that this competitive interaction may not occur in plants.

The kinetochore is essential for the equal segregation of chromosomes during cell division, a function vital for genome maintenance and conserved across eukaryotes from yeast to animals. However, its structure and components vary significantly among species. Although core kinetochore components in plants have been identified, the lack of molecular framework for these components has limited understanding. This study provides a comprehensive atlas of plant kinetochore factors along with their localization during the cell cycle (Fig. 7). Our findings suggest plant-specific kinetochore features significantly affect genome stability through SAC localization and broader functions beyond the M phase.

Inner kinetochore proteins, known as CCAN, exhibit low sequence homology but high structural conservation between yeast and human (Yan et al. 2019). In both, CCAN components CENP-C and CENP-T bind directly to the centromere, essential for CCAN assembly into kinetochores. However, some organisms, such as *C. elegans* and *D. melanogaster* have lost most CCAN components, including CENP-T, relying solely on CENP-C for chromatin binding (Singleton 2016). Arabidopsis similarly retains only CENP-C, CENP-S, CENP-X, and CENP-O, with only CENP-C localizing to the kinetochore and copurifying with other components (Figs. 1 and 2A to D). Similarly, in *P. patens* moss, only CENP-C has been observed at the kinetochore (Kozgunova et al. 2019). Thus, it is likely that chromosome-kinetochore binding via CENP-C is the primary pathway in plants, similar to *C. elegans* and *D. melanogaster*, with other CCANs have lost their kinetochore function in plant lineages. Despite our efforts, we were unable to detect CENH3 in our IP with CENP-C. Several potential explanations exist for this observation. First, CENH3 is a small protein enriched in arginine and lysine residues, which makes it highly susceptible to over-digestion by trypsin and complicates its identification by mass spectrometry. Another possibility is that kinetochores and chromosomes are stabilized by proteins other than CENP-C, such as CENP-T in vertebrates. It remains uncertain whether a similar factor exists in plant kinetochores (Fig. 7).

CENP-S and CENP-X were not localized to kinetochores in either *P. patens* moss or Arabidopsis (Kozgunova et al. 2019; Fig. 1), suggesting that these proteins have lost their canonical kinetochore function in plant lineages. Notably, in humans, CENP-S and CENP-X form a heterodimer involved not only in kinetochore assembly but also in DNA repair. Our results also demonstrate that CENP-S and CENP-X form heterodimers in Arabidopsis. Moreover, previous studies have reported their involvement in DNA repair in Arabidopsis (Girard et al. 2014), supporting the idea that while their kinetochore functions may be diminished, their role in DNA repair has been conserved. CENP-O also failed to localize kinetochores, and its mutant exhibited no obvious phenotypes. However, given its conservation across numerous plant species, it may retain an unrecognized function, akin to NNF1 (Allipra et al. 2022). Further studies will be necessary to uncover the potential roles of these atypical kinetochore components in plants.

The CCAN components, CENP-C and CENP-T, independently recruit the KMN network to kinetochores several organisms, including Xenopus, chicken, and humans. The MIS12C directly binds the N-terminal region of CENP-C (1 TO 71 aa in human; Screpanti et al. 2011), recruiting NDC80 and the other KMN elements. Concurrently, CENP-T binds directly to NDC80C, aiding KMN network (Nishino et al. 2013; Huisin’T Veld et al. 2016; Takenoshita et al. 2022). Although the N-terminal sequence of Arabidopsis CENP-C is less conserved compared to its human counterpart (Supplementary Fig. S8), Arabidopsis CENP-C copurifies with most KMN components, suggesting its role in KMN recruitment to the kinetochore. In fact, our BiFC assay demonstrated a direct interaction between CENP-C and DSN1, a subunit of the MIS12C. While it remains unclear whether this interaction is mediated specifically through the N-terminus of CENP-C, it likely plays a key role in establishing the connection between CCAN and KMN in Arabidopsis (Fig. 7).

In human cells, NDC80C's kinetochore localization involves SPC24 and SPC25 binding to DSN1 and NSL1 of MIS12C, and KNL1C binding to NSL1 via KNL1 (Petrovic et al. 2010). However, in Arabidopsis our Y2H results showNDC80C binds to MIS12 via NDC80 and SPC24. Furthermore, Arabidopsis KNL1 binds to MIS12 and NUF2 of NDC80C, indicating significant differences in KMN network construction compared to humans (Fig. 7). Protein alteration for kinetochore assembly is common; for example, DSN1 is absent in *Drosophila* but replaced functionally by Spc105R, a KNL1 homolog (Przewloka et al. 2009; Liu et al. 2016). Further kinetochore plasticity is observed in protozoan parasites like apicomplexans and kinetoplastids, which lack many known kinetochore components and have highly divergent kinetochores (Akiyoshi and Gull 2014; Brusini et al. 2022). This diversity suggests that understanding of kinetochore evolution requires data from a broad range of organisms.

We found that the KMN network construction and kinetochore formation timing vary between animals and among plant lineages. In human cells, MIS12C and KNL1 are recruited to the outer kinetochores during the S phase, with NDC80 released from the nucleus, restricting kinetochore formation to the M phase (Gascoigne and Cheeseman 2013). In the moss *P. patens*, NDC80 localizes to the kinetochores only during the M phase (Kozgunova et al. 2019). Conversely, in Arabidopsis, all KMN components localize to the kinetochores throughout the cell cycle, affecting kinetochore functions and the SAC localization potentially impacting SAC regulation (see further discussion below).

Our IP experiments indicate Arabidopsis core kinetochore factors may interact with previously unidentified noncanonical kinetochore proteins. CDKA;1, a key cell cycle regulatory kinase, was detected in most IP lists. We observed phosphorylation of KNL1, NUF2, and DSN1 at their CDK consensus sites, suggesting post-translational regulation by CDKA;1. In human cells, CDK1 phosphorylates kinetochore proteins like CENP-C and NSL1, enhancing their binding to CENP-A and KNL1, respectively (Watanabe et al. 2019; Lim-Manley et al. 2023). However, these specific amino acid residues are not conserved in plants, indicating different target kinetochore proteins.

Our IP data also identified proteins involved in DNA replication, including PCNA1, PCNA2, RFC3, RPA2, FEN1, and MCM3. PCNA1 and PCNA2 broadly copurified with core kinetochore proteins, while RFC3, RPA2, FEN1, and MCM3 mainly copurified with KMN network proteins. The continuous assembly of the Arabidopsis KMN network throughout the cell cycle may serve as a scaffold for these DNA replication factors.

Interestingly, NASP was also identified in the IP list. In plants, CENH3 (also known as CENP-A in mammals) chaperones analogous to HJURP in animals and Scm3 in fungi have not yet been identified. However, Le Goff et al. (2020) reported that NASP contributes to CENH3 loading in Arabidopsis. NASP is localized to the nucleoplasm during interphase (Le Goff et al. 2020; Takeuchi et al. 2024), and in our IP experiments, it was detected in samples pulled down with CENP-S, CENP-C, DSN1, and ZWINT1.1. Among these, DSN1 is the most likely candidate for direct interaction with NASP, given its shared nucleoplasmic localization. While the interaction between histone chaperones and kinetochore factors remains poorly understood, a recent study has shown that the FACT complex, another type of CENP-A chaperone, interacts with kinetochore factors in human cells (Schweighofer et al. 2025). Interestingly, our BiFC assay indicates an interaction between DSN1 and the inner kinetochore protein CENP-C. This raises the possibility that, upon CENP-A loading at the centromere, CENP-C may function as a scaffold for NASP.

Additionally, the IP list includes factors related to histone modification, chromatin remodeling, and microtubule-directed cell elongation, highlighting plant kinetochores' significant roles throughout the cell cycle. Future research should verify the physiological significance of these interactions.

The SAC is crucial for genome stability, ensuring equal chromosome segregations during cell division. In animals, MPS1's localization at the kinetochore, where it binds directly to NDC80, activates the SAC signal. We have previously shown that continuous stress inactivates the Arabidopsis SAC, leading to mitosis without nuclear division and resulting in polyploid cells (Komaki and Schnittger 2017). This plant-specific SAC regulation may impact plant evolution by affecting ploidy levels. The exact SAC regulatory mechanisms in Arabidopsis are unclear, but MPS1 localizes to the kinetochore throughout the cell cycle, unlike in animals (Komaki and Schnittger 2017). This study suggests that the Arabidopsis KMN network is consistently directed to the kinetochore, which may explain the persistent localization of MPS1. Therefore, the phosphorylation activity of MPS1, rather than its localization, is the primary SAC regulatory mechanism in plants. Interestingly, in the moss *P. patens* both MPS1 and the KMN network localize to the kinetochore only during the M phase (Kozgunova et al. 2019), indicating differences in SAC regulation between mosses and flowering plants. Moreover, the scaffold proteins responsible for the recruitment of MPS1 also appear to differ between animals and plants. In yeast and human cells, MPS1 and microtubules compete for binding to NDC80, and displacement of MPS1 by microtubule attachment is a key mechanism for SAC silencing (Hiruma et al. 2015; Ji et al. 2015). In contrast, in Arabidopsis, MPS1 signals persist into anaphase (Komaki and Schnittger 2017), suggesting an alternative silencing pathway. Additionally, our findings imply that MPS1 binds directly to KNL1 but not to NDC80 (Fig. 4A), raising the possibility that SAC regulation in plants differs from that in other eukaryotes. However, this interpretation remains preliminary, and additional studies, such as in vitro binding assays, will be important to clarify the molecular mechanisms underlying SAC silencing in plants.

Previous studies and our findings reveal that in monocots, KNL1 binds to BMF1 and BMF2, while in dicots, it binds to BMF3 (Fig. 4A; Su et al. 2021; Deng et al. 2024). In yeast and animals, KNL1's conserved MELT motif, phosphorylated by MPS1, interacts with BUB1, analogous to BMF1 (Caldas and DeLuca 2014). However, since plant KNL1 lacks a conserved MELT motif (Tromer et al. 2015), it is crucial to investigate if MPS1 activity is required for KNL1-BMFs binding. We also found Arabidopsis BMF1 binds to CENP-C. Remarkably, *M. polymorpha*'s sole BMF-like protein binds both KNL1 and CENP-C, hinting that the function of BMF may have split into multiple proteins during plant evolution. Although the last eukaryotic common ancestor (LECA) likely had a single BUB1 family protein (referred to as MadBub; Suijkerbuijk et al. 2012), no MadBub-CENP-C interactions are reported. Thus, the BMF-CENP-C interaction might be plant specific. Further biological and bioinformatics studies are needed to verify this hypothesis.

In conclusion, our analysis highlights plant-specific interactions between kinetochore components and kinetochore-SAC components in Arabidopsis. These intriguing interactions were supported by multiple approaches, including protein localization, IP, Y2H, and BiFC assays. Given that all GFP-tagged lines were introduced into a wildtype background where endogenous proteins are present, it remains uncertain whether their function is entirely preserved. Moreover, the possibility that endogenous proteins may compete with GFP-tagged counterparts could impede the detection of weak or transient interactions during IP. Therefore, additional studies, such as in vitro binding assays and genetic analyses using mutant lines, are essential to validate these interactions and clarify their physiological significance. We also discovered that core kinetochore components interact with factors functioning outside of M phase, suggesting that plant kinetochores might operate throughout the cell cycle. The universality of these characteristics across plants and their role in genome plasticity remain unclear due to limited studies on plant kinetochores. However, a recent report found key kinetochore and SAC components missing in *Cuscuta* genus parasitic plants, suggesting dynamic rearrangements in plant kinetochores (Neumann et al. 2023). Further research is needed to understand plant-specific SAC regulation and its potential impact on genome plasticity.

## Materials and methods

### Plant materials and growth conditions

The *A. thaliana* accession Columbia (Col-0) was used as the wildtype in this study. Plants were grown on a solid medium containing half-strength Murashige and Skoog (MS) salts, 1% (w/v) sucrose and 1.5% (w/v) agar in a growth chamber (16 h day/8 h night at 22 °C).

The T-DNA insertion line SALK_149069 (*cenp-o-1*) was obtained from the Arabidopsis Biological Resource Center (ABRC). Primer pairs for genotyping are described in Supplementary Table S5 and Supplementary Fig. S1.

### Plasmid construction and transgenic plants

To create kinetochore markers, the genomic fragment of each kinetochore gene was amplified with *attB* sites by PCR and cloned into *pDONR221*. The *Sma*I site (*Nae*I site for *NNF1*) was inserted in front of the start codon (*CENP-C* and *NDC80*) or the stop codon (the other genes) of the constructs. The resulting construct was linearized by *Sma*I digestion and was ligated to the monomeric enhanced *GFP* (*mEGFP*) gene, followed by LR recombination reactions with the destination vector pGWB501 (Nakagawa et al. 2007). To create the CRISPR/CAS9 construct against the *CENP-O* gene, a *CENP-O*-gene-specific spacer sequence was cloned into the pEn-Sa-Chimera, followed by LR recombination reaction with the destination vector pDe-Sa-CAS9 (Steinert et al. 2015). Primer pairs for plasmid construction are listed in Supplementary Table S5. Transgenic plants were generated by the floral dip method (Clough and Bent 1998). The constructs were introduced into *Agrobacterium tumefaciens* strain GV3101 (pMP90) and used for plant transformation.

### Immunoprecipitation

150 to 200 transgenic seedlings were harvested and flash-frozen in liquid nitrogen for 2 min. The total proteins from seedlings were extracted following the described method, with certain modifications (Magyar et al. 1997). Briefly, tissue homogenization was performed using a TissueLyser (Qiagene) (50 Hz, 4 × 30 s) with 6 mm stainless steel beads and sterile sand. Samples were then resuspended in 800 µL of RIPA lysis buffer supplemented with protease and phosphatase inhibitors, as well as the proteasome inhibitor MG132. The complete composition of the lysis buffer was: 150 mm NaCl, 1% IGEPAL CA-630, 0.5% sodium deoxycholate, 0.1% SDS, 50 mm Tris-HCl (pH 8.0), 1 mm DTT, 1 mm PMSF, 1× Sigma Protease Inhibitor Cocktail, 3 mm  *p*-nitrophenylphosphate (pNPP), and 1 µM MG132. Lysates were centrifuged at 16,000 × *g* for 15 min at 4 °C. The supernatant was incubated with 50 µL of Miltenyi anti-GFP bead matrix for 20 min with a method modified from (Hubner et al. 2010; Horvath et al. 2017). The beads were then sequentially washed: twice with RIPA buffer, five times with PBS, and once with ABC buffer. Proteins bound to the beads were reduced with DTT and alkylated with iodoacetamide (IAM). Trypsin digestion was carried out for 2 h at 47 °C. The reaction was acidified following digestion, and one-sixth of each sample was loaded onto EvoTips for LC-MS analysis.

### Mass spectrometry

The digestion mixtures were acidified and transferred to a single-use trapping mini-column (Evotip; 1/8 of the samples) and then analyzed with a data-dependent LC-MS/MS method using an Evosep One (LC: 15 SPD; MS1: R:120,000) on-line coupled to a linear ion trap-Orbitrap (Orbitrap-Fusion Lumos, Thermo Fisher Scientific) mass spectrometer operating in positive ion mode. Data acquisition was carried out in data-dependent fashion, multiply charged ions were selected in cycle-time from each MS survey scan for ion-trap HCD fragmentation (MS spectra were acquired in the Orbitrap (R = 60,000) while MSMS in the ion-trap).

### Data interpretation

Raw data were converted into peak lists using the in-house Proteome Discoverer (v 1.4) (Guan et al. 2011) and searched against the Swissprot database (downloaded 2019/6/12, 560,292 proteins) using the Protein Prospector search engine (v5.15.1) with the following parameters: enzyme: trypsin with maximum 1 missed cleavage; mass accuracies: 5 ppm for precursor ions and 0.6 Da for fragment ions (both monoisotopic); fixed modification: carbamidomethylation of Cys residues; variable modifications: acetylation of protein N-termini; Met oxidation; cyclization of N-terminal Gln residues allowing maximum 2 variable modifications per peptide. Acceptance criteria: minimum scores: 22 and 15; maximum E values: 0.01 and 0.05 for protein and peptide identifications, respectively. Another database search was also performed using the same search and acceptance parameters except that Uniprot.random.concat database (downloaded 2022/07/20) was searched with *A. thaliana* species restriction (136,466 proteins) including additional proteins identified from the previous Swissprot search (protein score > 50).

### Confocal microscopy

The root tips of 5-day-old seedlings were utilized for live cell imaging. Samples were placed in glass-bottom dishes and covered with a solid medium containing half-strength MS salts, 1% sucrose, and 1.5% agar. Confocal images were acquired using a Leica TCS SP8 inverted confocal microscope with a HC PL APO 63×/1.20 CS2 water immersion objective. mEGFP and TagRFP-T were excited with 488 nm (laser intensity: 80%) and 555 nm (laser intensity: 10%) lasers, respectively. Emission was collected at 493 to 550 nm for mEGFP and 577 to 650 nm for TagRFP-T, with detector gain values set to 150 and 100, respectively. Images were obtained at 20-second intervals with four repetitions of line averaging and corrected for image drift by the StackReg plugin (Rigid Body option) for ImageJ version 1.54f.

### Yeast two-hybrid assay

To create the Yeast two-hybrid (Y2H) constructs, the cDNA fragments of each gene were amplified by PCR and cloned into *pENTR4* by the SLiCE method or amplified with *attB* sites by PCR and cloned into *pDONR221*. The *MPS1*, *BMF3*, *MAD1*, and *MAD2* constructs were generated by previously (Komaki and Schnittger 2017). The subcloned cDNAs were subsequently integrated into the *pGBT9-GW* (DNA-BD) or *pGAD424-GW* (AD) vectors by LR recombination reactions. The resulting constructs were transformed into the yeast strain AH109. Transformants were spotted onto control (–TL), moderately selective (–TLH), and severely selective (–TLHA) media and photographed after incubation at 30 °C for 3 days. Primer pairs for plasmid construction are described in Supplementary Table S5.

### Generation of the network in Cytoscape

Protein–protein interaction networks were visualized using Cytoscape (version 3.10.1; Shannon et al. 2003). The network was constructed from interaction data obtained by IP and/or Y2H assays. The node attributes were defined according to protein features. The color of each node indicates the subcomplex assignment of the corresponding protein, and the node size reflects the relative frequency of its occurrence. Edge attributes were assigned based on the experimental method by which the interaction was detected. Interactions identified exclusively by IP are shown in green, those detected exclusively by Y2H are shown in blue, and interactions supported by both assays are indicated in red.

### BiFC assay

To change the antibiotic resistance of the plasmids, the BiFC cassette of each Multicolor BiFC Gateway plasmid (Gehl et al. 2009) was amplified by PCR and inserted into the plasmid carrying the spectinomycin resistance gene by the SLiCE method. The resulting constructs were designated as *pDEST-GW:nYFP*, *pDEST-GW:cYFP*, *pDEST-nYFP:GW*, and *pDEST-cYFP:GW*. The targeted cDNAs and *GUS* cDNA (used as a negative control for BiFC), were subsequently introduced into the modified *pDEST* vectors via LR recombination reactions. For the centromere marker, the TagRFP in the TagRFP:CENH3 construct (Komaki and Schnittger 2017) was replaced with mScarlet-I. Primer pairs for plasmid construction are listed in Supplementary Table S5.

These constructs were transformed into *A. tumefaciens* strain GV3101 (pMP90). For transient expression, mixtures of different BiFC-partner strains and the p19 helper strain were coinfiltrated into 4- to 5-week-old *N. benthamiana* leaves. Fluorescence signals were examined for 3 days postinoculation. Confocal images were acquired using a Leica TCS SP8 inverted confocal microscope with a HC PL APO 20×/0.75 CS2 dry objective. mVenus and mScarlet-I were excited using 513 and 569 nm lasers, respectively, both at 80% laser intensity. Emission was collected at 518 to 560 nm for mVenus and 600 to 650 nm for mScarlet-I, with detector gain values set to 100. Images were acquired with four repetitions of line averaging.

### Accession numbers

Sequence data from this article can be found under the following accession numbers: *BMF1* (At2g20635), *BMF2* (At2g33560), *BMF3* (At5g05510), *BUB3.1* (At3g19590), *BUB3.2* (At1g49910), *BUB3.3* (At1g69400), *MAD1* (At5g49880), *MAD2* (At3g25980), *MPS1* (At1g77720). *MpBMF* (Mapoly0009s0056), *MpCENP-C* (Mapoly0008s0161), and *MpKNL1* (Mapoly0030s0102). The accession numbers of Arabidopsis core kinetochore genes are listed in Table 1.

## Supplementary Material

kiaf461_Supplementary_Data

## Data Availability

The data underlying this article are available in the article and its online Supplementary material.

## Supplementary data

The following materials are available in the online version of this article.

**Supplementary Figure S1.** Phenotypic analysis of cenp-o mutants.

**Supplementary Figure S2.** Y2H assay: Interaction between Arabidopsis core kinetochore components.

**Supplementary Figure S3.** Y2H assay: Interaction between core kinetochore and SAC components.

**Supplementary Figure S4.** BiFC assay: Interaction between MPS1 and scaffold proteins.

**Supplementary Figure S5.** Y2H assay: Interaction among Arabidopsis SAC components.

**Supplementary Figure S6.** Y2H assay: Interaction between KNL1 and its interactors.

**Supplementary Figure S7.** Structural prediction of KNL1 using AlphaFold2 and 3.

**Supplementary Figure S8.** Sequence alignment of the N-terminus of CENP-C.

**Supplementary Table S1.** Immunoprecipitated protein list.

**Supplementary Table S2.** Results of GO enrichment analysis.

**Supplementary Table S3.** List of interactors with core kinetochore components in Arabidopsis.

**Supplementary Table S4.** Identified phosphopeptides of kinetochore proteins.

**Supplementary Table S5.** Primers used in this study.

**Supplementary Video S1.** Subcellular localization of CENP-S:GFP during mitosis.

**Supplementary Video S2.** Subcellular localization of CENP-X:GFP during mitosis.

**Supplementary Video S3.** Subcellular localization of CENP-O:GFP during mitosis.

**Supplementary Video S4.** Subcellular localization of GFP: CENP-C during mitosis.

**Supplementary Video S5.** Subcellular localization of GFP:NDC80 during mitosis.

**Supplementary Video S6.** Subcellular localization of NUF2:GFP during mitosis.

**Supplementary Video S7.** Subcellular localization of SPC24.1:GFP during mitosis.

**Supplementary Video S8.** Subcellular localization of SPC24.2:GFP during mitosis.

**Supplementary Video S9.** Subcellular localization of SPC25:GFP during mitosis.

**Supplementary Video S10.** Subcellular localization of MIS12:GFP during mitosis.

**Supplementary Video S11.** Subcellular localization of DSN1:GFP during mitosis.

**Supplementary Video S12.** Subcellular localization of NNF1:GFP during mitosis.

**Supplementary Video S13.** Subcellular localization of NSL1.1:GFP during mitosis.

**Supplementary Video S14.** Subcellular localization of KNL1:GFP during mitosis.

**Supplementary Video S15.** Subcellular localization of ZWINT1.1:GFP during mitosis.
