# Supplementary material for: Functional framework of the kinetochore and spindle assembly checkpoint in Arabidopsis
Source: Plant Physiol. 2025 Sep 30;199(2):kiaf461. doi: 10.1093/plphys/kiaf461 (PMC12526889; doi:10.1093/plphys/kiaf461)
Supplement: kiaf461_Supplementary_Data [file kiaf461_supplementary_data.zip › Supplementary Figure S1-S8_final.pdf]

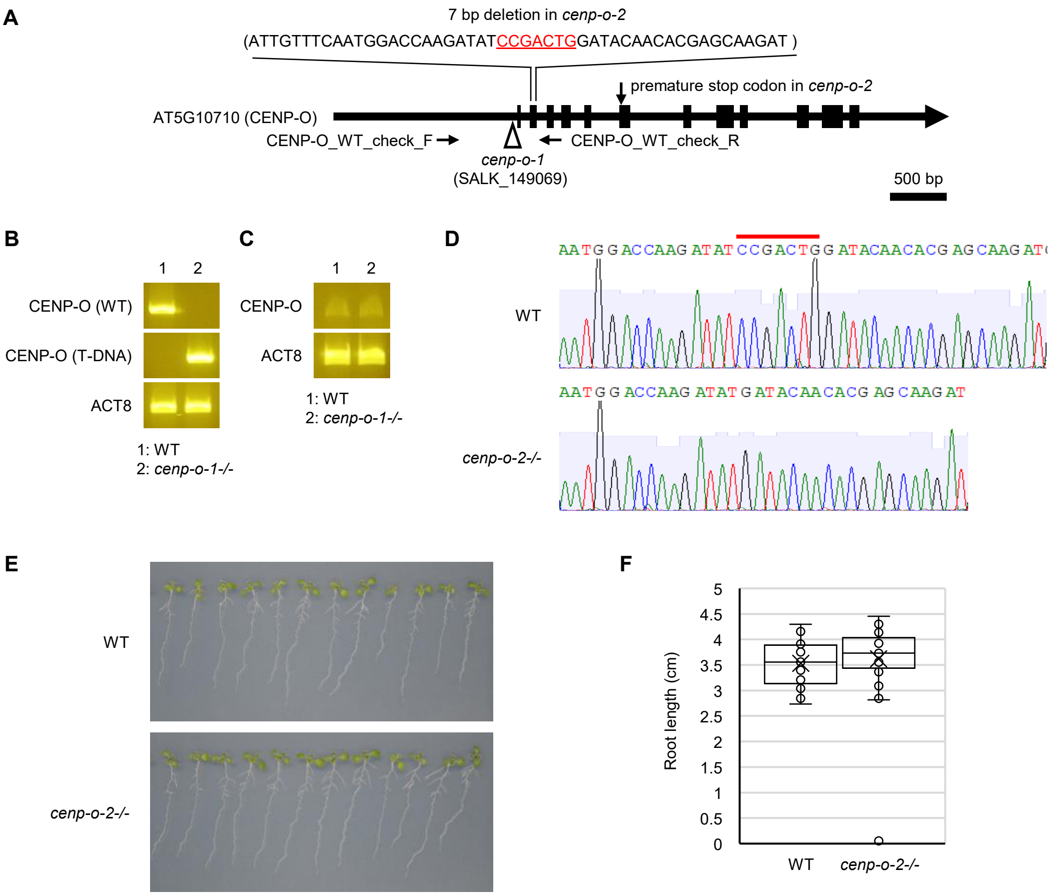

Supplementary Figure S1. Phenotypic analysis of *cenp-o* mutants. A, Gene structure of the *CENP-O* gene in Arabidopsis. Arrowhead indicates the T-DNA insertion site in *cenp-o-1*. Red color indicates the deleted nucleotides in *cenp-o-2*. B, Genotyping of *cenp-o-1* by PCR. ACT8 is used as a control. C, Expression analysis of *CENP-O* full-length cDNA in *cenp-o-1* by RT-PCR. ACT8 is used as a control. D, Sequence analysis of *cenp-o-2*. E, Seven-day-old seedlings grown on vertically placed plates. F, Root length of 7-day-old seedlings. The box plot shows the median (center line) and the first and third quartiles (box boundaries). Whiskers extend to the minimum and maximum values, and individual data points, including outliers, are plotted as dots. No statistically significant difference was observed by Student's t-test (n = 39). Primer pairs are described in Supplementary Table S4.

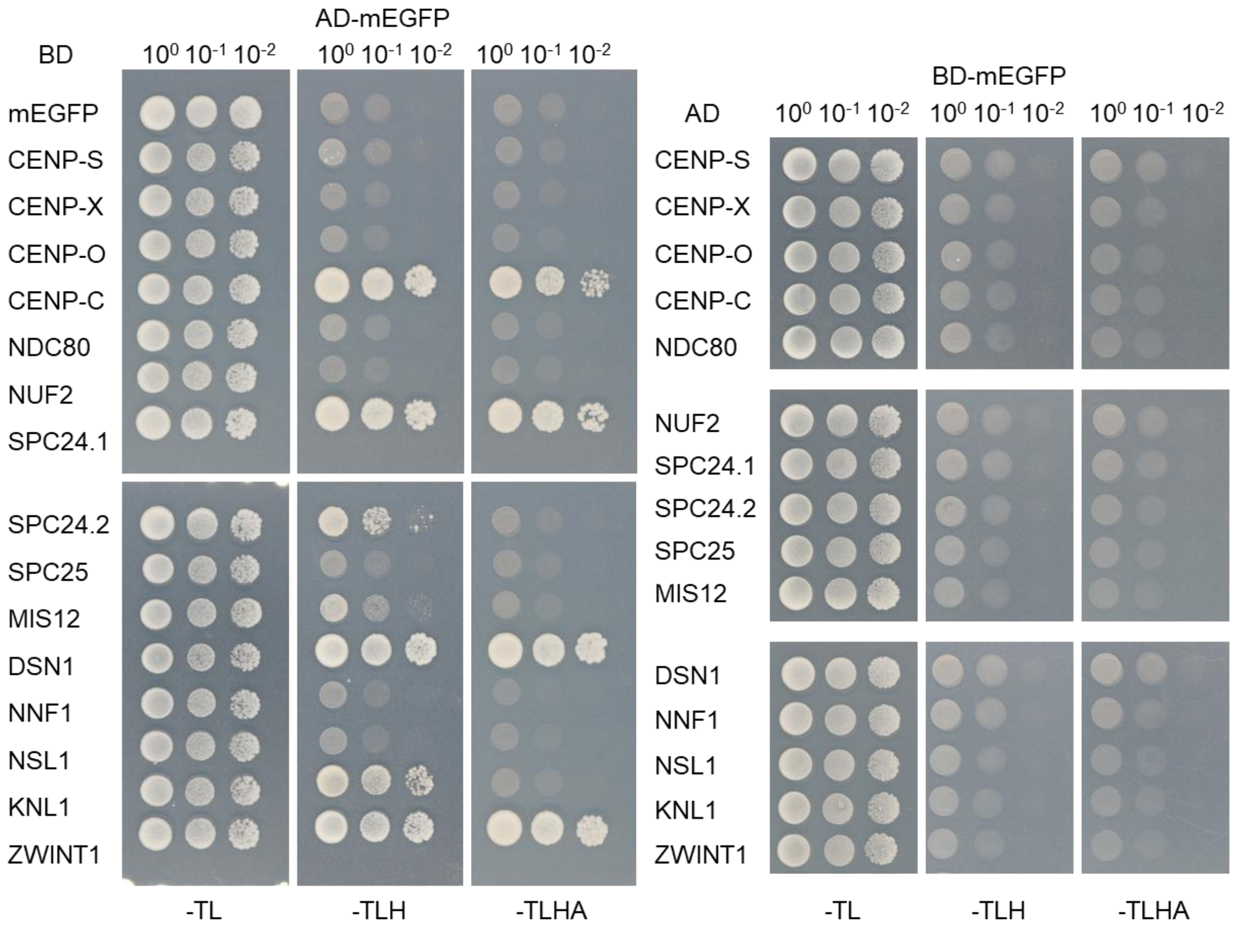

AD-mEGFP
BD
10^0 10^-1 10^-2
mEGFP
CENP-S
CENP-X
CENP-O
CENP-C
NDC80
NUF2
SPC24.1
SPC24.2
SPC25
MIS12
DSN1
NNF1
NSL1
KNL1
ZWINT1
-TL
-TLH
-TLHA
BD-mEGFP
AD
CENP-S
CENP-X
CENP-O
CENP-C
NDC80
NUF2
SPC24.1
SPC24.2
SPC25
MIS12
DSN1
NNF1
NSL1
KNL1
ZWINT1
-TL
-TLH
-TLHA

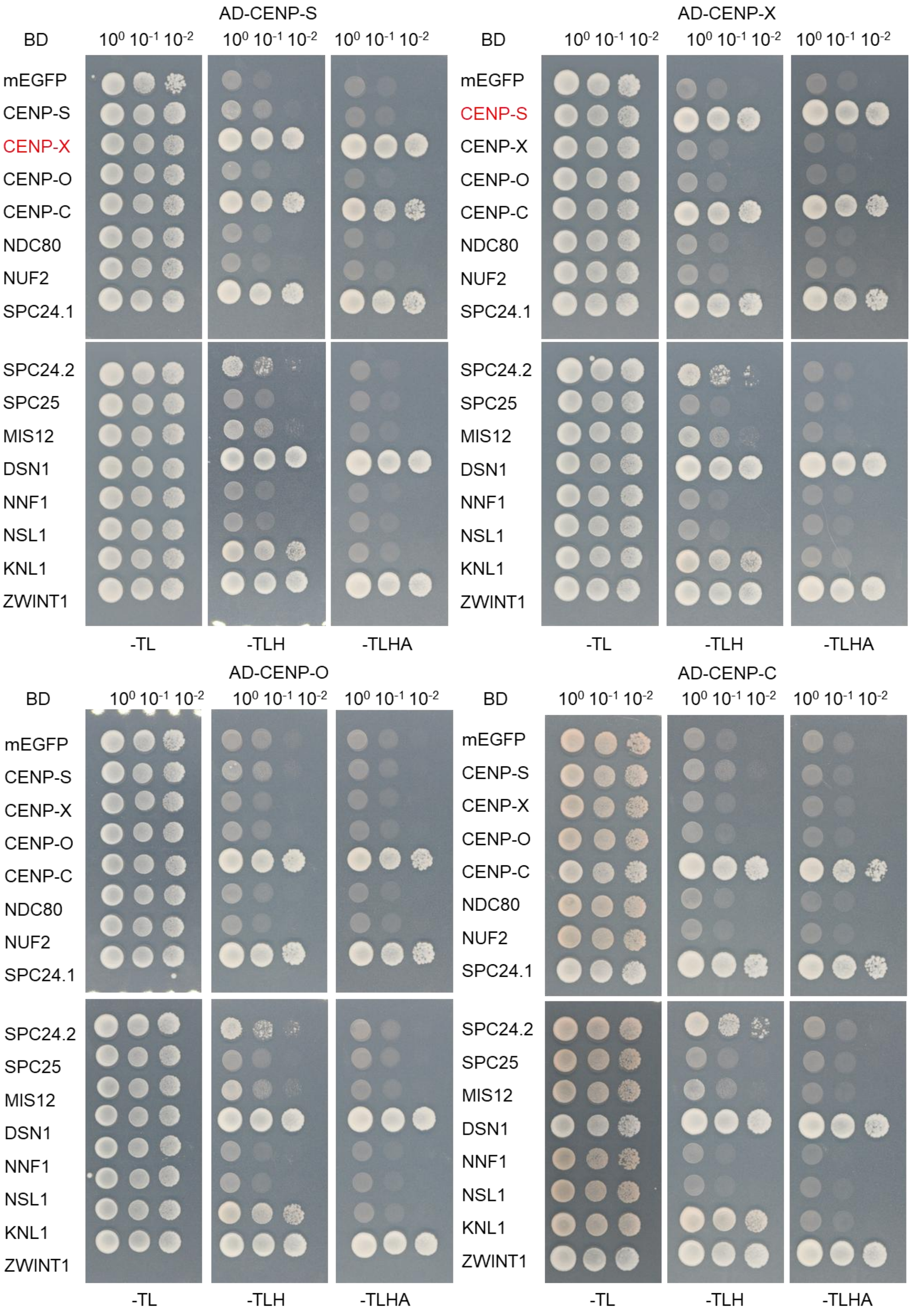
AD-CENP-S
AD-CENP-X
AD-CENP-O
AD-CENP-C
BD
10^0 10^{-1} 10^{-2}
mEGFP
CENP-S
CENP-X
CENP-O
CENP-C
NDC80
NUF2
SPC24.1
SPC24.2
SPC25
MIS12
DSN1
NNF1
NSL1
KNL1
ZWINT1
-TL
-TLH
-TLHA

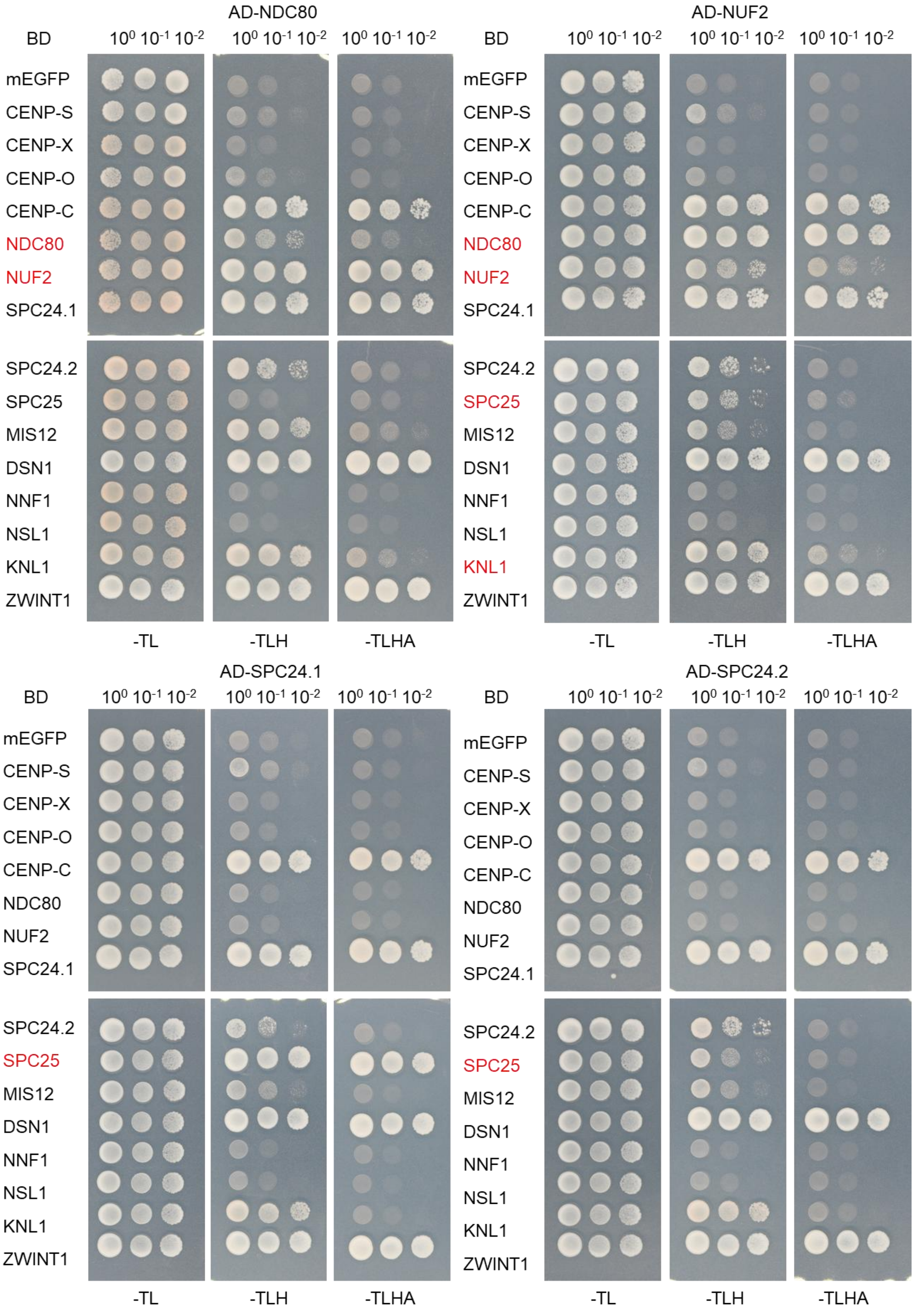
AD-NDC80
AD-NUF2
AD-SPC24.1
AD-SPC24.2
BD
10^0 10^-1 10^-2
mEGFP
CENP-S
CENP-X
CENP-O
CENP-C
NDC80
NUF2
SPC24.1
SPC24.2
SPC25
MIS12
DSN1
NNF1
NSL1
KNL1
ZWINT1
-TL
-TLH
-TLHA

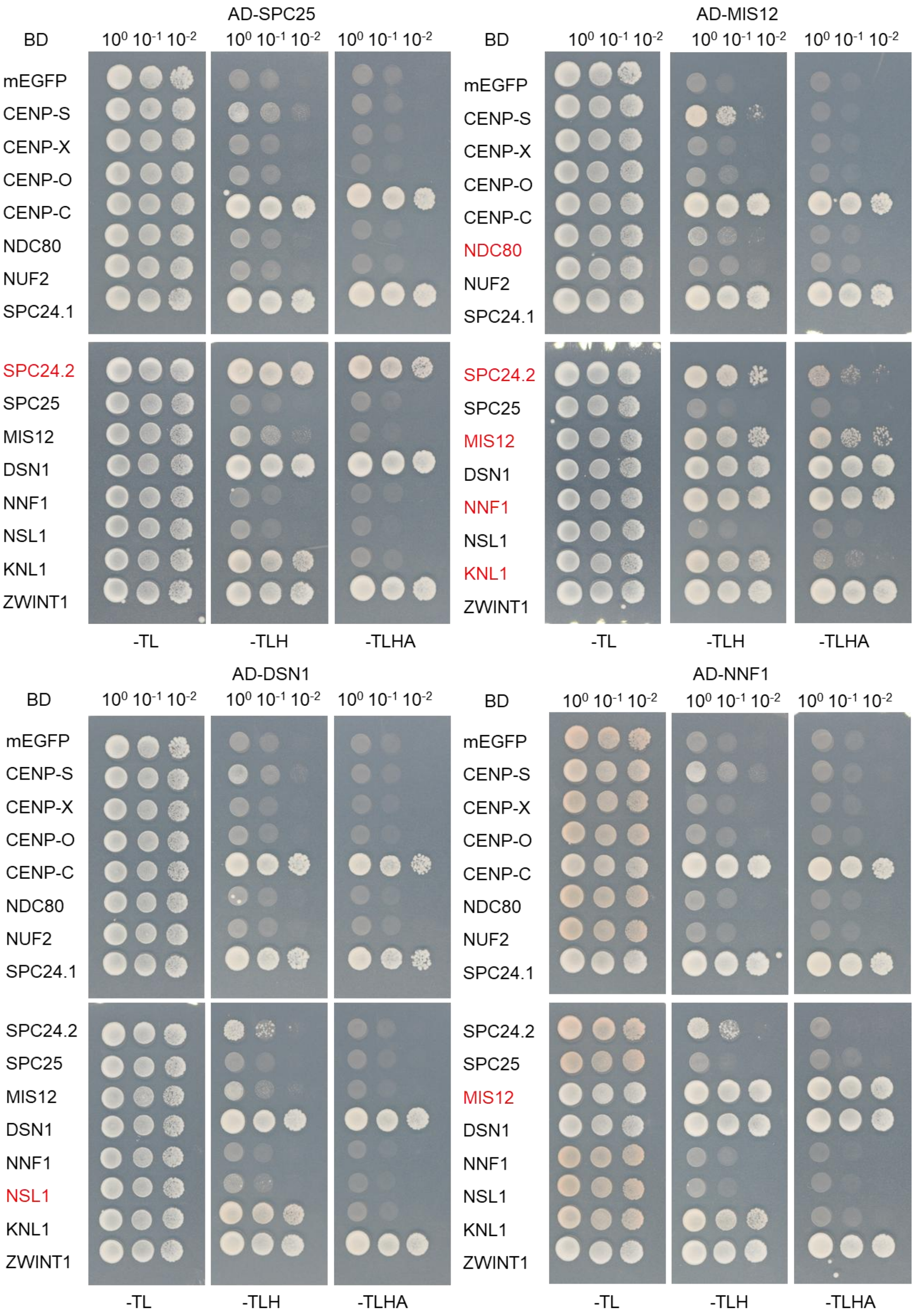
AD-SPC25
BD
10^{0} 10^{-1} 10^{-2}
mEGFP
CENP-S
CENP-X
CENP-O
CENP-C
NDC80
NUF2
SPC24.1
SPC24.2
SPC25
MIS12
DSN1
NNF1
NSL1
KNL1
ZWINT1
-TL
-TLH
-TLHA
AD-MIS12
BD
10^{0} 10^{-1} 10^{-2}
mEGFP
CENP-S
CENP-X
CENP-O
CENP-C
NDC80
NUF2
SPC24.1
SPC24.2
SPC25
MIS12
DSN1
NNF1
NSL1
KNL1
ZWINT1
-TL
-TLH
-TLHA
AD-DSN1
BD
10^{0} 10^{-1} 10^{-2}
mEGFP
CENP-S
CENP-X
CENP-O
CENP-C
NDC80
NUF2
SPC24.1
SPC24.2
SPC25
MIS12
DSN1
NNF1
NSL1
KNL1
ZWINT1
-TL
-TLH
-TLHA
AD-NNF1
BD
10^{0} 10^{-1} 10^{-2}
mEGFP
CENP-S
CENP-X
CENP-O
CENP-C
NDC80
NUF2
SPC24.1
SPC24.2
SPC25
MIS12
DSN1
NNF1
NSL1
KNL1
ZWINT1
-TL
-TLH
-TLHA

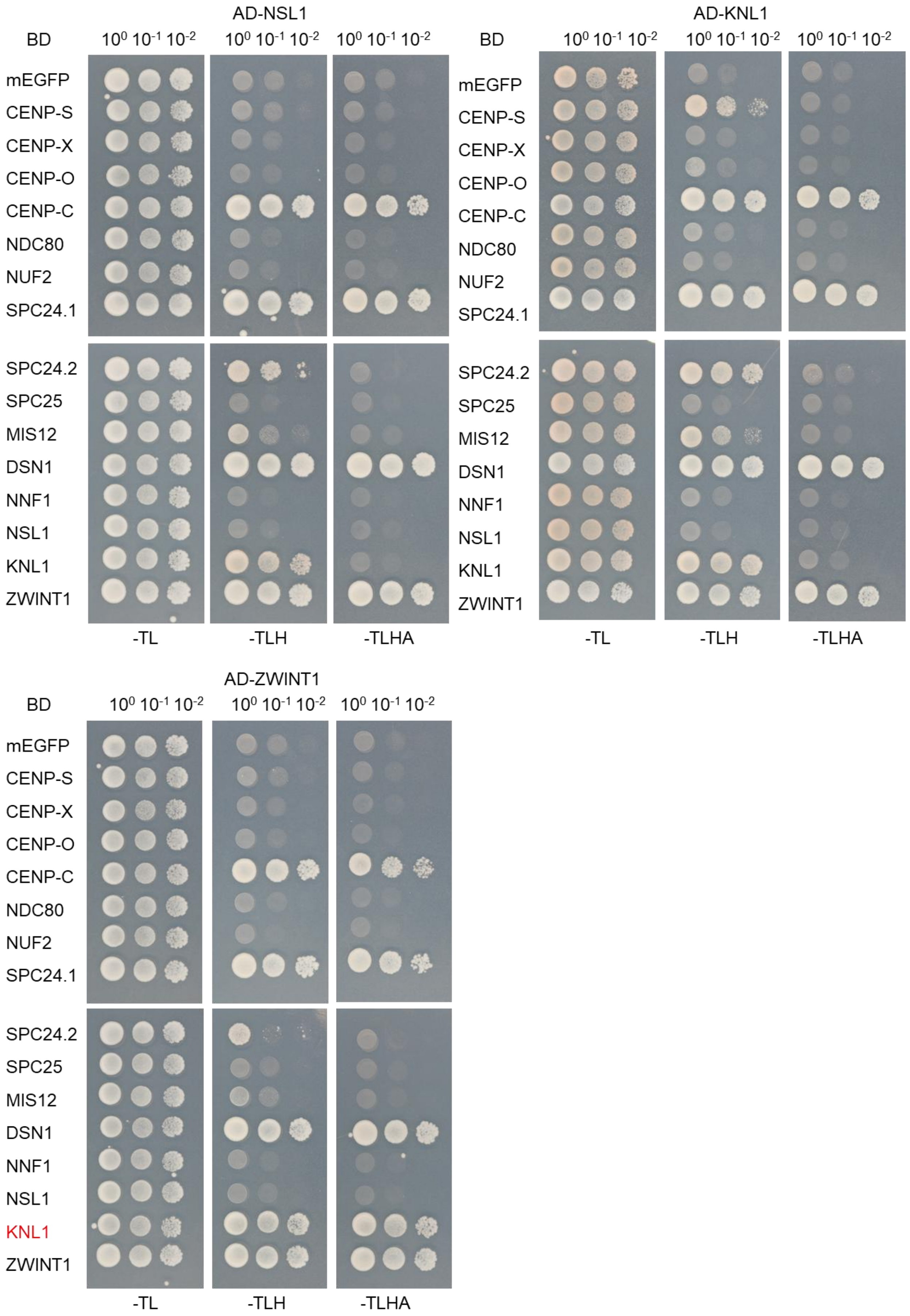
AD-NSL1
BD
10^0 10^{-1} 10^{-2}
mEGFP
CENP-S
CENP-X
CENP-O
CENP-C
NDC80
NUF2
SPC24.1
SPC24.2
SPC25
MIS12
DSN1
NNF1
NSL1
KNL1
ZWINT1
-TL
-TLH
-TLHA
AD-KNL1
BD
10^0 10^{-1} 10^{-2}
mEGFP
CENP-S
CENP-X
CENP-O
CENP-C
NDC80
NUF2
SPC24.1
SPC24.2
SPC25
MIS12
DSN1
NNF1
NSL1
KNL1
ZWINT1
-TL
-TLH
-TLHA
AD-ZWINT1
BD
10^0 10^{-1} 10^{-2}
mEGFP
CENP-S
CENP-X
CENP-O
CENP-C
NDC80
NUF2
SPC24.1
SPC24.2
SPC25
MIS12
DSN1
NNF1
NSL1
KNL1
ZWINT1
-TL
-TLH
-TLHA

Supplementary Figure S2. Interactions between core kinetochore components were detected by Y2H assay. Monomeric enhanced GFP (mEGFP) was used as a negative control. Each strain was spotted on SD plates without tryptophan and leucine (TL; control media) or without tryptophan, leucine, and histidine (TLH; moderately selective media) or without tryptophan, leucine, histidine, and adenine (TLHA; severely selective media) and photographed after incubation at 30˚C for three days. AD, activating domain; BD, DNA-binding domain. Red color combinations indicate that the yeast growth are more vigorous than mEGFP controls.

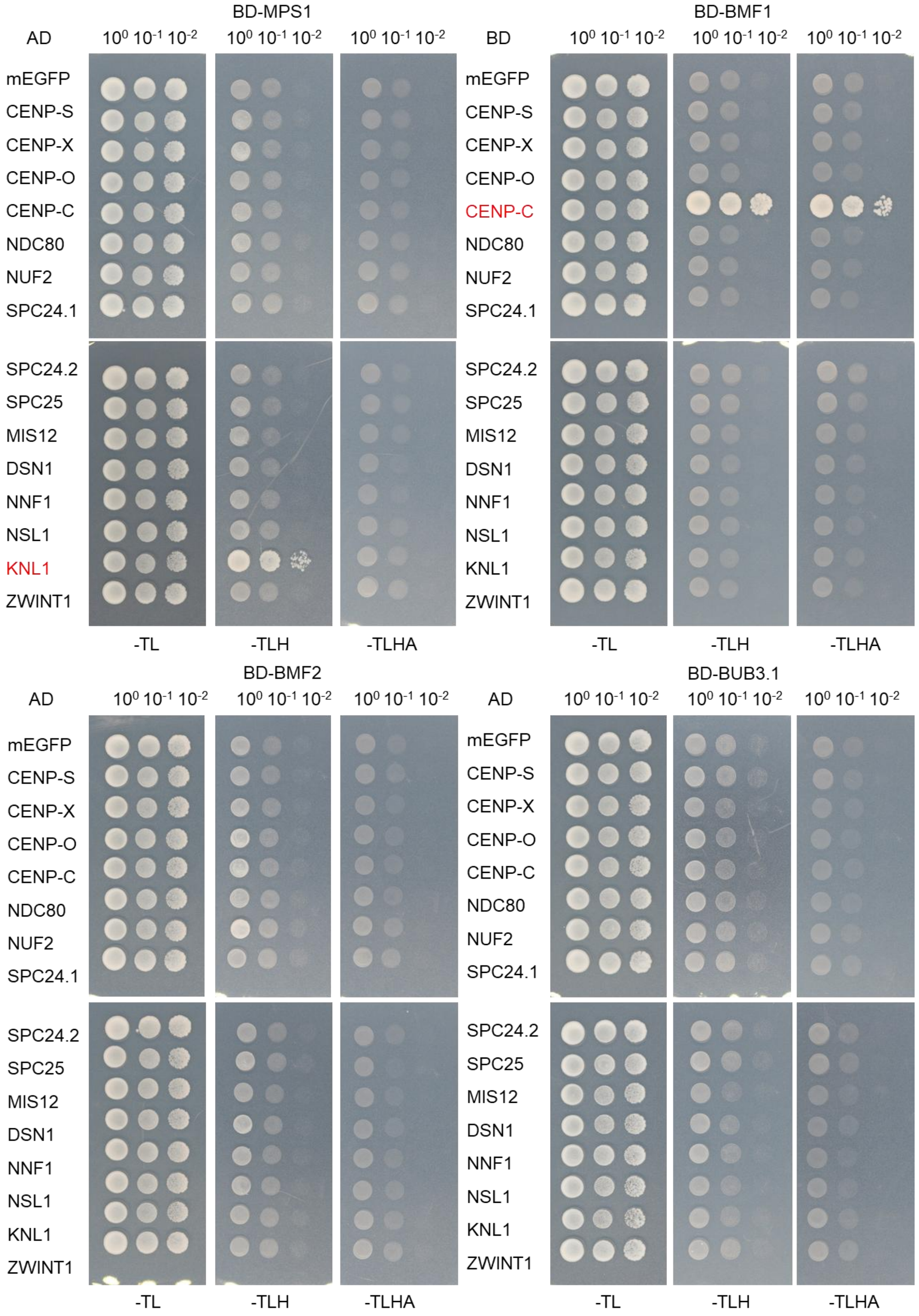
BD-MPS1
AD
10^0 10^{-1} 10^{-2}
BD
BD-BMF1
mEGFP
CENP-S
CENP-X
CENP-O
CENP-C
NDC80
NUF2
SPC24.1
SPC24.2
SPC25
MIS12
DSN1
NNF1
NSL1
KNL1
ZWINT1
-TL
-TLH
-TLHA
BD-BMF2
BD-BUB3.1
AD

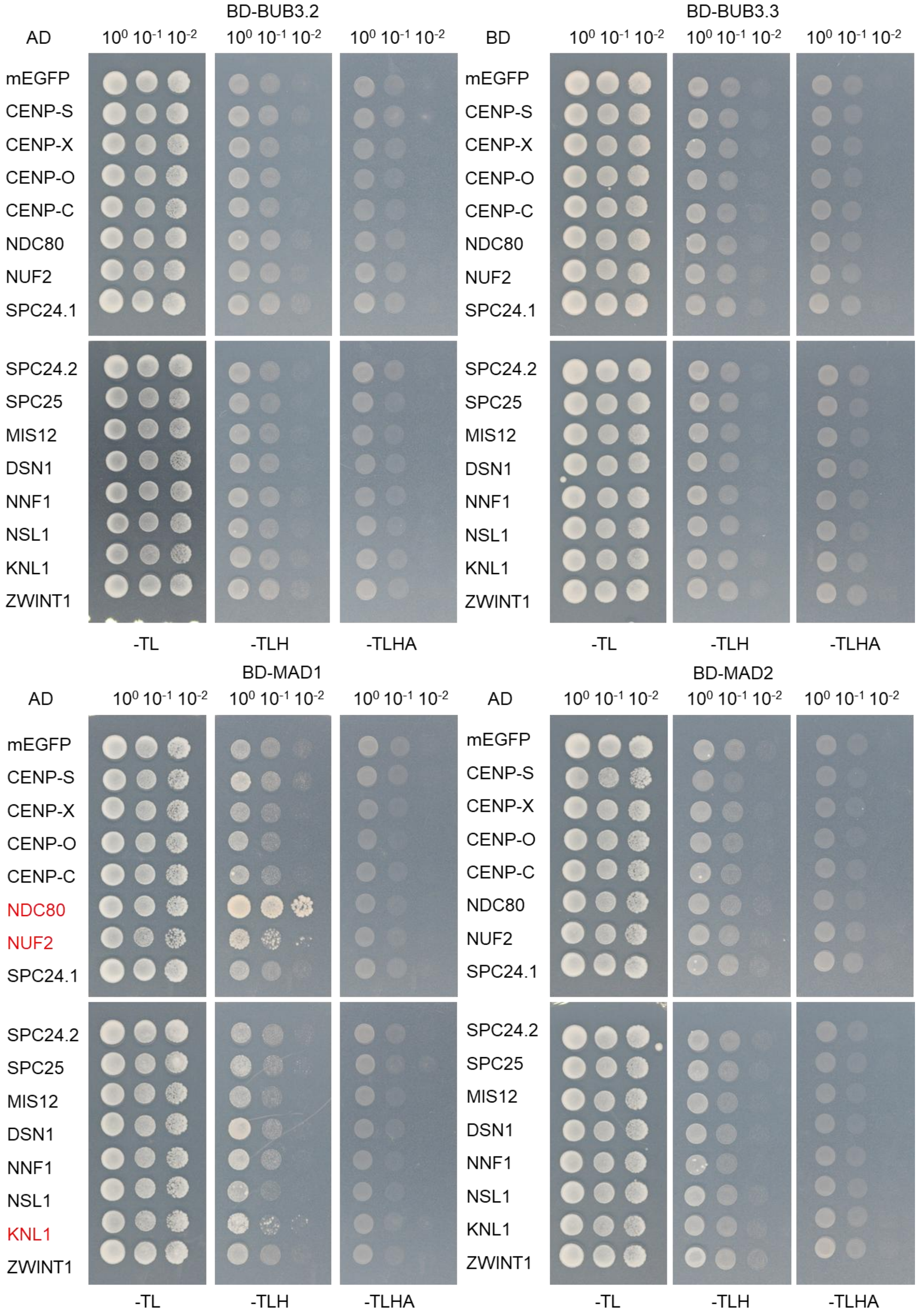
BD-BUB3.2
AD
10^0 10^-1 10^-2
mEGFP
CENP-S
CENP-X
CENP-O
CENP-C
NDC80
NUF2
SPC24.1
SPC24.2
SPC25
MIS12
DSN1
NNF1
NSL1
KNL1
ZWINT1
-TL
-TLH
-TLHA
BD-BUB3.3
BD
BD-MAD1
BD-MAD2

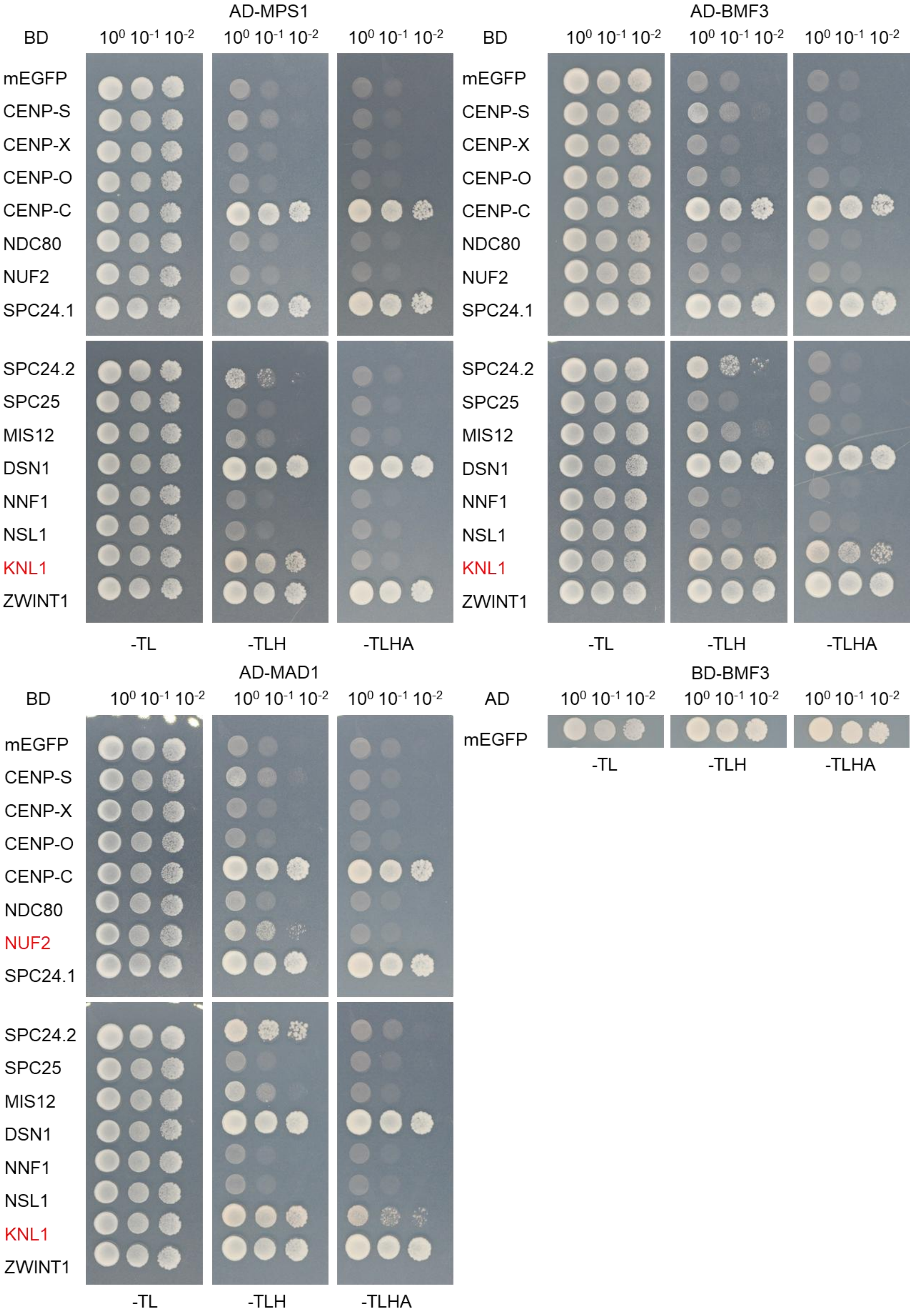
AD-MPS1
BD
10^0 10^{-1} 10^{-2}
mEGFP
CENP-S
CENP-X
CENP-O
CENP-C
NDC80
NUF2
SPC24.1
SPC24.2
SPC25
MIS12
DSN1
NNF1
NSL1
KNL1
ZWINT1
-TL
-TLH
-TLHA
AD-BMF3
AD-MAD1
BD-BMF3
AD
mEGFP
-TL
-TLH
-TLHA

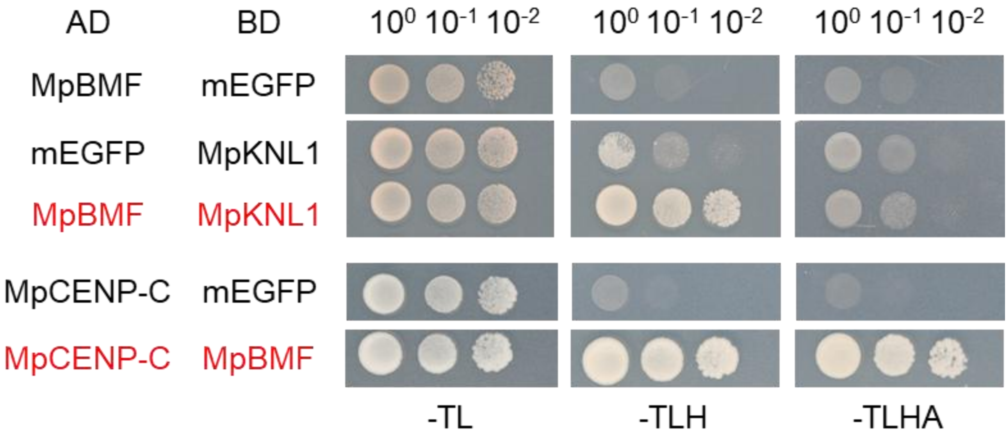

Supplementary Figure S3. Interactions between core kinetochore components and SAC components were detected by Y2H assay. Monomeric enhanced GFP (mEGFP) was used as a negative control. Each strain was spotted on SD plates without tryptophan and leucine (TL; control media) or without tryptophan, leucine, and histidine (TLH; moderately selective media) or without tryptophan, leucine, histidine, and adenine (TLHA; severely selective media) and photographed after incubation at 30˚C for three days. AD, activating domain; BD, DNA-binding domain. Red color combinations indicate that the yeast growth are more vigorous than mEGFP controls.

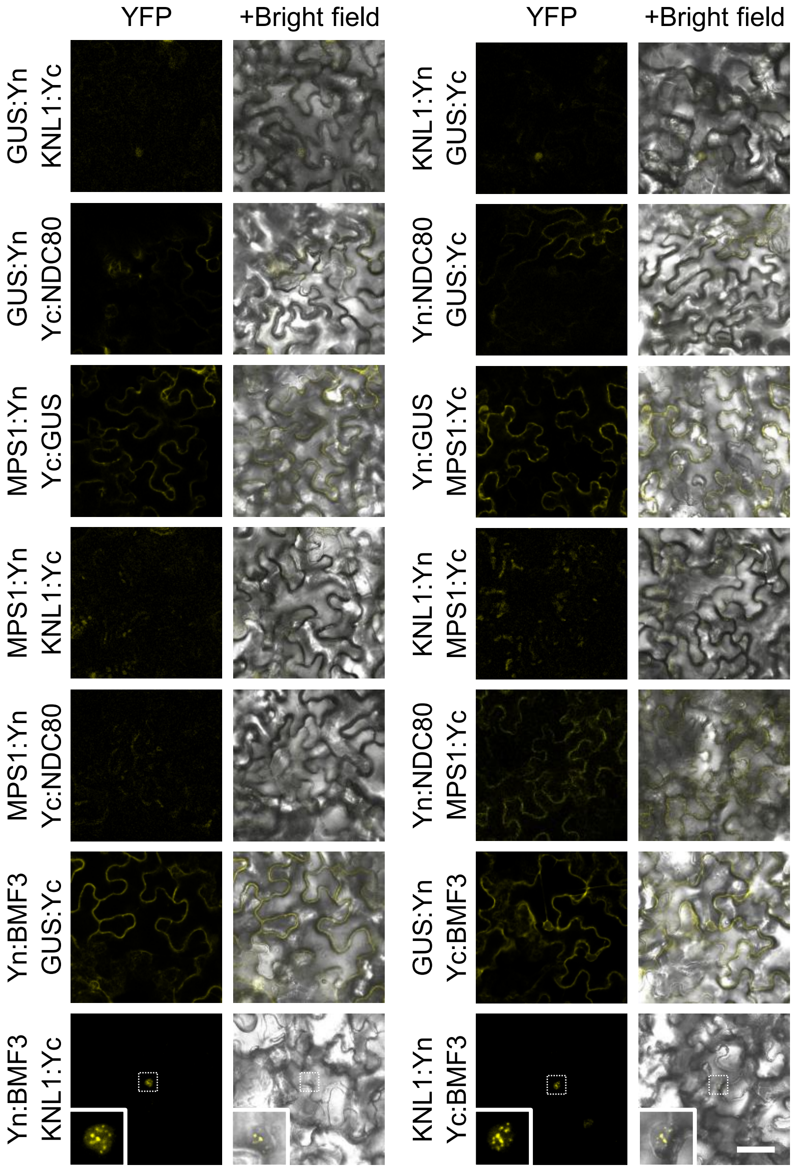

Supplementary Figure S4. Interaction between MPS1 and scaffold proteins. Analysis of interactions between MPS1 and candidate scaffold proteins in *Nicotiana benthamiana* by BiFC assay. Proteins were fused to either the N-terminal (Yn) or C-terminal (Yc) fragment of YFP. The GUS protein served as a negative control, while BMF3 served as a positive control for interaction with KNL1. Boxed regions are shown at higher magnification (4x) to highlight YFP fluorescence signals. Scale bar, 50 μm.

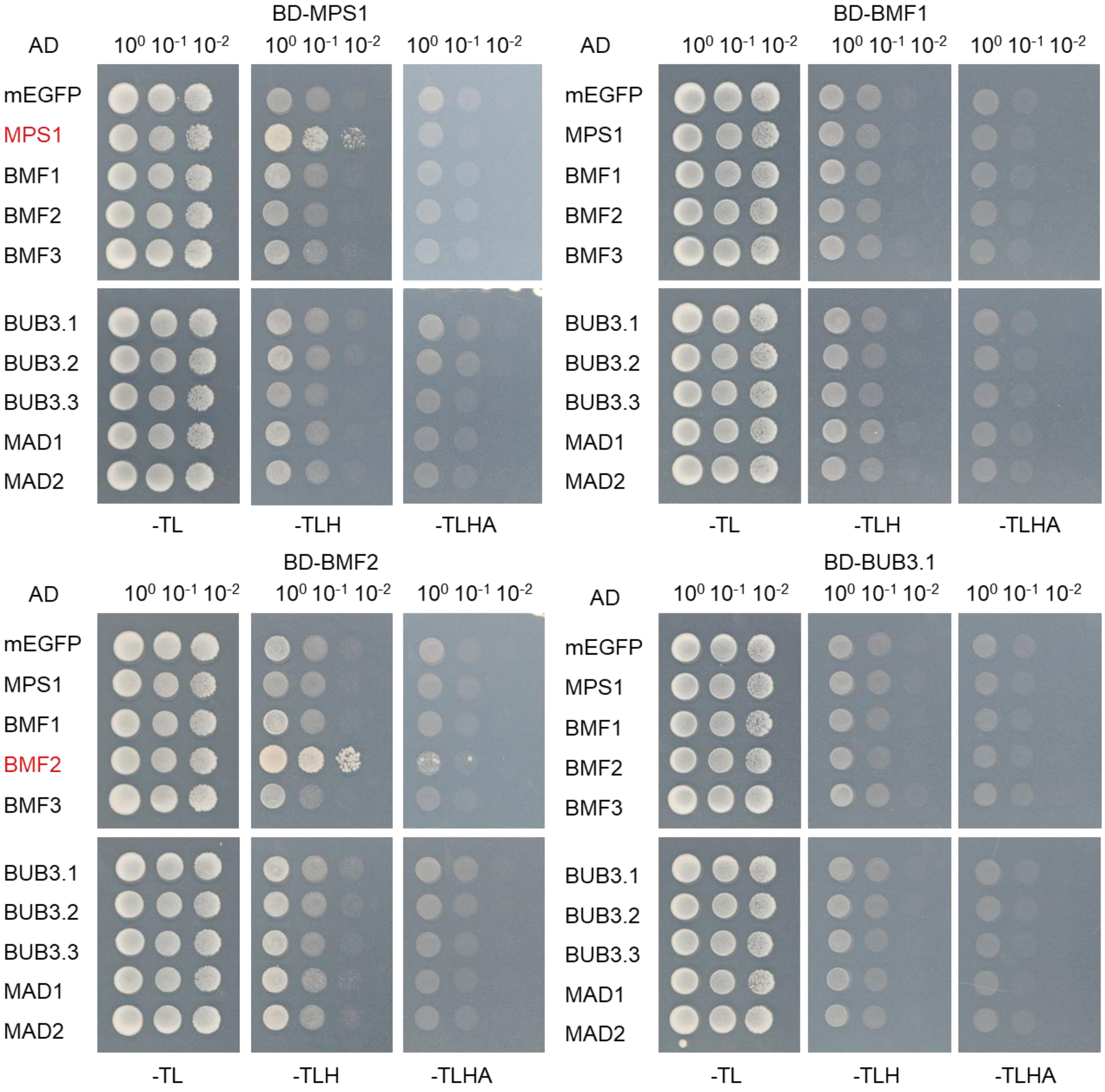
BD-MPS1
AD
10⁰ 10⁻¹ 10⁻²
mEGFP
MPS1
BMF1
BMF2
BMF3
BUB3.1
BUB3.2
BUB3.3
MAD1
MAD2
-TL
-TLH
-TLHA
BD-BMF1
BD-BMF2
BD-BUB3.1

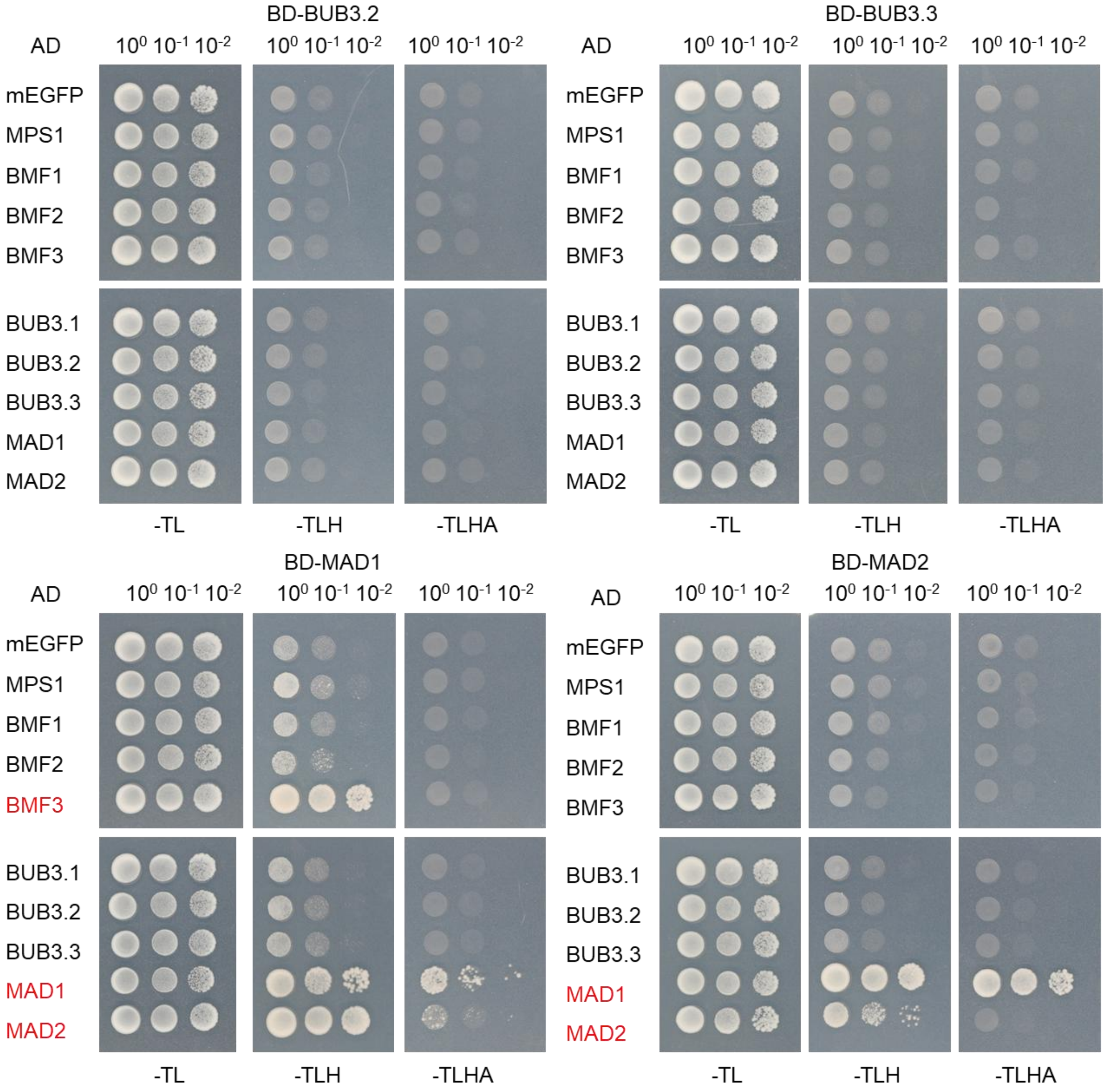

Supplementary Figure S5. Interactions between SAC kinetochore were detected by Y2H assay. Monomeric enhanced GFP (mEGFP) was used as a negative control. Each strain was spotted on SD plates without tryptophan and leucine (TL; control media) or without tryptophan, leucine, and histidine (TLH; moderately selective media) or without tryptophan, leucine, histidine, and adenine (TLHA; severely selective media) and photographed after incubation at 30˚C for three days. AD, activating domain; BD, DNA-binding domain. Red color combinations indicate that the yeast growth are more vigorous than mEGFP controls.

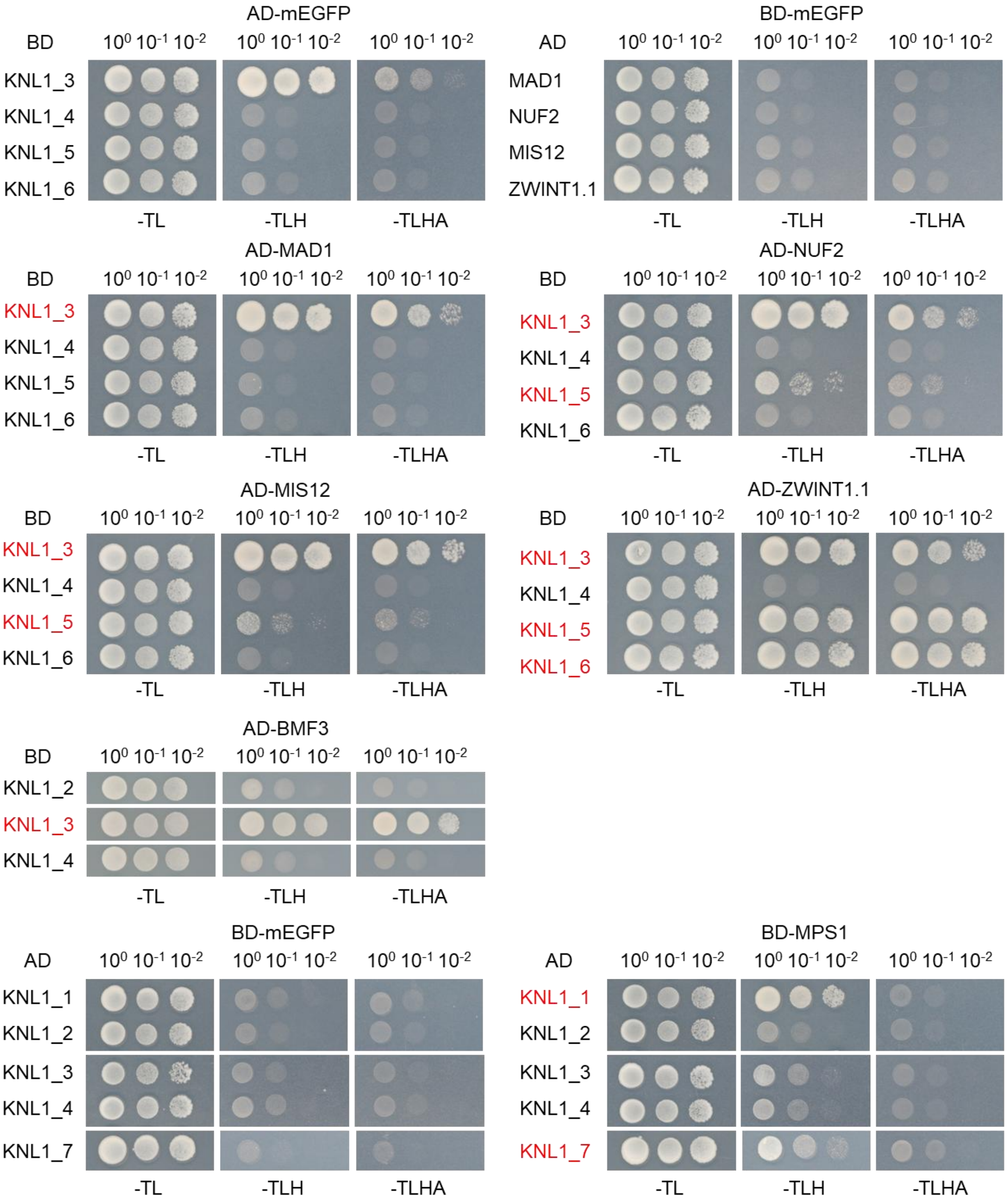

Supplementary Figure S6. Interaction domain of KNL1 with each interactor were detected by Y2H assay. Monomeric enhanced GFP (mEGFP) was used as a negative control. Each strain was spotted on SD plates without tryptophan and leucine (TL; control media) or without tryptophan, leucine, and histidine (TLH; moderately selective media) or without tryptophan, leucine, histidine, and adenine (TLHA; severely selective media) and photographed after incubation at 30˚C for three days. AD, activating domain; BD, DNA-binding domain. Red color combinations indicate that the yeast growth are more vigorous than mEGFP controls.

A CLUSTAL O(1.2.4) multiple sequence alignment (KNL1)

```
AT2G04235             LVESLSTIQKSKSRLGLIPPSPG-SAL----------SQRIEKSKLQLSGHRFLTTPSIG
Solyc07g045170.3      PWTSSASLQKSISKLPFLEDPI--SFLSNGKRGPWTSSASLQKSISKLERLKASAFSSFG
Glyma.13G008500       HGATPSSVHRSILKMKTLETTPVMSGL----------KEGMDRLKARLSK----YSPGFS
                         : :::::*  ::  :      * *          .  :::   :*.        .:.

AT2G04235             REEIGVLRDKHADIPITNLEALLSKHDNRTPISEEKSMPDKCISGALSHAVD 450
Solyc07g045170.3      GDKIPHMGVRALEFPKTPPLDSILK----------KRNLDMGVKRLDAAMTC 561
Glyma.13G008500       LSNIKDREYKQDESRQTPLEEKLFSL-------TPDSNVHKGLVDSNDHGIQ 471
                       .:*     :  :   *     : .          .   .  :
```

B UniProt ID: F4IV62

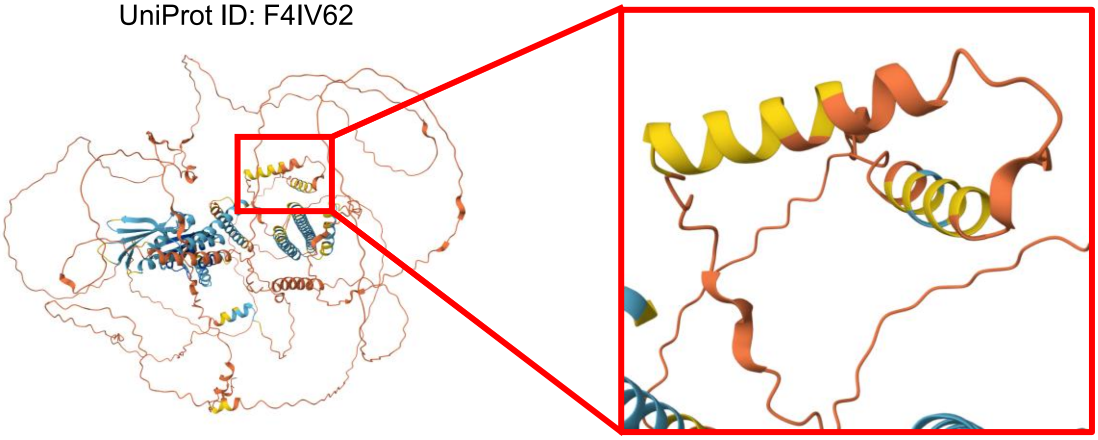

A. thaliana KNL1: 1-1227 aa
(From AlphaFold Protein Structure Database)

A. thaliana KNL1: 350-450 aa

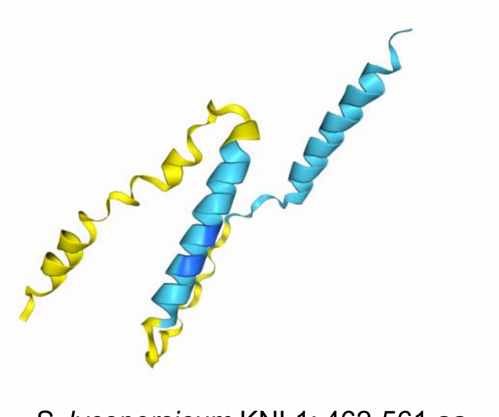

S. lycopersicum KNL1: 462-561 aa
(Predicted by AlphaFold 3)

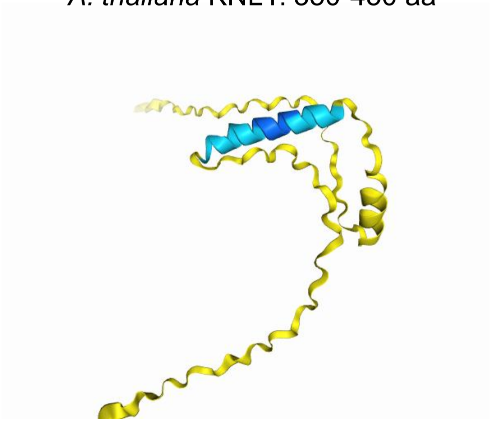

G. max KNL1: 381-471 aa
(Predicted by AlphaFold 3)

Supplementary Figure S7. Multiple sequence alignments and protein structure analysis. A, Protein sequence of 350 to 450 aa of Arabidopsis KNL1 (AT2G04235) and the corresponding region of tomato KNL1 (Solyc07g045170.3) and soybean KNL1 (Glyma.13G008500) are aligned using Clustal Omega (https://www.ebi.ac.uk/jdispatcher/msa/clustalo). B, Protein structure of 350 to 450 aa of Arabidopsis KNL1 and the corresponding region of tomato KNL1 and soybean KNL1 are obtained from AlphaFold Protein Structure Database (https://alphafold.ebi.ac.uk/) or predicted by AlphaFold 3 (https://alphafoldserver.com/).

CLUSTAL O(1.2.4) multiple sequence alignment (CENP-C)

```
H. sapiens      MAASGLDHLKNGYRRRFCRPSRARDINTEQGQNVLEILQDCFEEKSLANDFSTNSTKSVP
A. thaliana     ----------------MADVSRSSSLYTEED----------------PLQAYSG--LSLFP
                               :.  **: .: **:.                 : :*    . .*

H. sapiens      NSTRKIKDTCI   71
A. thaliana     RTLKSL-----   33
                .: :.:
```

Supplementary Figure S8. Multiple sequence alignments. Protein sequence of 1 to 71 aa of Human CENP-C (NP_001803.2) and the corresponding region of Arabidopsis CENP-C are aligned using Clustal Omega (https://www.ebi.ac.uk/jdispatcher/msa/clustalo).
