# Supplementary material for: Functional framework of the kinetochore and spindle assembly checkpoint in Arabidopsis
Source: Plant Physiol. 2025 Sep 30;199(2):kiaf461. doi: 10.1093/plphys/kiaf461 (PMC12526889; doi:10.1093/plphys/kiaf461)
Supplement: kiaf461_Supplementary_Data [file kiaf461_supplementary_data.zip › Supplementary Video Legends.docx]

**Supplementary Video Legends**

**Supplementary Video S1. Subcellular localization of CENP-S:GFP during mitosis.**

**Supplementary Video S2. Subcellular localization of CENP-X:GFP during mitosis.**

**Supplementary Video S3. Subcellular localization of CENP-O:GFP during mitosis.**

**Supplementary Video S4. Subcellular localization of GFP: CENP-C during mitosis.**

**Supplementary Video S5. Subcellular localization of GFP:NDC80 during mitosis.**

**Supplementary Video S6. Subcellular localization of NUF2:GFP during mitosis.**

**Supplementary Video S7. Subcellular localization of SPC24.1:GFP during mitosis.**

**Supplementary Video S8. Subcellular localization of SPC24.2:GFP during mitosis.**

**Supplementary Video S9. Subcellular localization of SPC25:GFP during mitosis.**

**Supplementary Video S10. Subcellular localization of MIS12:GFP during mitosis.**

**Supplementary Video S11. Subcellular localization of DSN1:GFP during mitosis.**

**Supplementary Video S12. Subcellular localization of NNF1:GFP during mitosis.**

**Supplementary Video S13. Subcellular localization of NSL1.1:GFP during mitosis.**

**Supplementary Video S14. Subcellular localization of KNL1:GFP during mitosis.**

**Supplementary Video S15. Subcellular localization of ZWINT1.1:GFP during mitosis.**

Supplementary Videos S1–S15 show the spatiotemporal localization of core kinetochore components in Arabidopsis roots. Root tips from five-day-old seedlings were used for live-cell imaging. Samples were mounted in glass-bottom dishes and covered with solid medium consisting of half-strength MS salts, 1% sucrose, and 1.5% agar. Confocal images were acquired using a Leica TCS SP8 inverted confocal microscope with a HC PL APO 63x/1.20 CS2 water immersion objective. mEGFP and TagRFP-T were excited with 488 nm (laser intensity: 80%) and 555 nm (laser intensity: 10%) lasers, respectively. Emission was collected at 493-550 nm for mEGFP and 577-650 nm for TagRFP-T, with detector gain values set to 150 and 100, respectively. Images were obtained at 20-second intervals with four repetitions of line averaging and corrected for image drift by the StackReg plugin (Rigid Body option) for ImageJ version 1.54f.
